# Virtual prevention of eating disorders in children, adolescents, and emerging adults: a scoping review

**DOI:** 10.1186/s40337-022-00616-8

**Published:** 2022-07-06

**Authors:** Danielle Pellegrini, Laura Grennan, Neera Bhatnagar, Gail McVey, Jennifer Couturier

**Affiliations:** 1 McMaster University, 1200 Main Street West, Hamilton, ON L8N 3Z5 Canada; 2 University Health Network, Toronto, ON Canada; 3 University of Toronto, Toronto, ON Canada; 4 McMaster Children’s Hospital, Hamilton, Canada

**Keywords:** Eating disorders, Prevention, Early intervention, Virtual, Children, Adolescents, Emerging adults

## Abstract

**Background:**

During the COVID-19 pandemic, there was a necessity for eating disorder (ED) outpatient treatment to be delivered virtually. Given this transition, and the surge in new ED cases, there was an urgent need to investigate virtually delivered ED prevention programs. This review aimed to identify the available evidence on virtual ED prevention programs for children, adolescents, and emerging adults.

**Method:**

Using scoping review methodology, seven databases were searched for studies published from January 2000 to April 2021 reporting on virtually delivered ED prevention interventions for children and adolescents (< 18 years) and emerging adults (18–25 years). Studies were excluded if they contained adults (> 25 years) and individuals with clinical ED diagnoses. Abstracts and full-text papers were reviewed independently by two reviewers. Data was extracted on study type, methodology, age, sample size, virtual intervention, outcomes, and results. In April 2022, we used a forward citation chaining process to identify any relevant articles from April 2021 to April 2022.

**Results:**

Of 5129 unique studies identified, 67 met eligibility criteria, which included asynchronous (n = 35) and synchronous (n = 18) internet-based programs, other e-technology including mobile apps (n = 3) and text messaging interventions (n = 1), computer-based programs (n = 6), and online caregiver interventions focused on child outcomes (n = 4). Few studies mainly included children and adolescents (n = 18), whereas the vast majority included emerging adults (n = 49). For children and adolescents, the most widely researched programs were *Student Bodies* and its adapted versions (n = 4), *eBody Project* (n = 2), and *Parents Act Now* (n = 2). For emerging adults, the most widely researched programs were *Student Bodies* and its adapted versions (n = 16), *eBody Project* (n = 6) and *Expand Your Horizon* (n = 4). These interventions were effective at reducing various symptoms and ED risk. Some studies demonstrated that virtual prevention intervention efficacy resembled in-person delivery.

**Conclusion:**

Virtual prevention interventions for EDs can be effective, however more research is needed studying their impact on children and adolescents and on improving access for vulnerable groups. Additional efficacy studies are required, such as for text messaging and mobile app ED prevention interventions. Evidence-based recommendations for virtual ED prevention for children, adolescents, and emerging adults at-risk for EDs should be prioritized.

**Supplementary Information:**

The online version contains supplementary material available at 10.1186/s40337-022-00616-8.

## Plain English summary

This review aimed to identify all available evidence for virtual eating disorder (ED) prevention interventions for children/adolescents (<18 years) and emerging adults (18-25 years). We reviewed seven databases and found 67 studies for inclusion. Findings were summarized into themes: asynchronous (not in real-time) and synchronous (in real-time) internet-based programs, other e-technology (mobile applications ['apps'], text messages), computer-based programs, and online caregiver interventions focused on child outcomes. Among children and adolescents, the most widely researched programs were *Student Bodies *(asynchronous internet-based cognitive-behavioural program), *eBody Project* (synchronous internet-based cognitive-dissonance program), and *Parents Act Now, *(online caregiver intervention). Among emerging adults, the most widely researched programs were *Student Bodies* (described above), *eBody Project* (described above) and *Expand Your Horizon* (asynchronous internet-based body functionality program). These interventions were effective at reducing symptoms and/or risk of developing EDs. Additional research is needed, including a greater focus on children and adolescents, and text messaging, mobile apps, online cognitive restructuring, and online imagery rescripting ED prevention interventions. Evidence-based recommendations for virtual ED prevention interventions that have been reviewed by a panel and research on improving access to virtual ED prevention services for vulnerable groups should also be prioritized.

**Supplementary Information:**

The online version contains supplementary material available at 10.1186/s40337-022-00616-8.

## Introduction

Eating disorders (EDs) cause significant impairment in mental and physical health-related quality of life, [1] and are potentially life-threatening, as they have one of the highest mortality rates of all psychiatric illnesses [2]. Known for being complex, chronic, and difficult to treat, especially if treatment is not pursued within the first three years of symptom onset [3], ED prevention interventions are warranted—particularly among vulnerable youth. Initial ED prevention programs were mostly psychoeducational, integrated into health curriculums, and taught in classroom settings, but no meaningful impact on ED risk factors was demonstrated [4]. After decades of research, it is now known that the most effective preventative interventions for EDs are multi-sessional and interactive, target high-risk individuals, and focus on specific risk factors for EDs, such as body dissatisfaction and thin-ideal internalization [5]. As technology has evolved, ED prevention programs followed suit, being delivered virtually as well as in-person. Past systematic review and meta-analysis research examined e-therapy and other e-mental health interventions for the prevention and treatment of EDs, with evidence suggesting virtually delivered programs can be effective in reducing ED risk factors and symptoms, such as shape/weight concerns, dietary restraint, ED psychopathology, drive for thinness, and thin-ideal internationalization; however, the reviews included a mix of youth and adult populations [6, 7]. A specific focus on virtual prevention for EDs among youth is warranted, especially during the COVID-19 pandemic.

During the COVID-19 pandemic, there has been a dramatic increase in the number of youth suffering from EDs (particularly Anorexia Nervosa) and requiring hospitalization [8]. Waitlists for outpatient treatment have nearly doubled, and programs are struggling to accommodate the growth in new ED cases [9]*.* To follow social distancing regulations, outpatient ED treatment around the world rapidly transitioned to virtual delivery. Our team recently published virtual care recommendations for children, adolescents, and emerging adults during the COVID-19 pandemic and beyond [10]; however, these did not include recommendations for virtual prevention for EDs. With the transition to predominantly virtual outpatient treatment delivery, as well as the surge in new ED cases among youth, it is imperative to evaluate and implement evidence-based, virtually delivered ED prevention programs.

The main aims of this scoping review were to identify the types of available evidence on virtual ED prevention programs for youth, specifically child, adolescent, and emerging adult populations, to summarize best practices in virtual prevention offerings, as well as to identify knowledge gaps in this field, prior to and during the COVID-19 pandemic.

## Methods

### Overview

Given the aims of our study, we used scoping review methodology [11–14] to ensure we collated a variety of evidence on virtual prevention for EDs for children, adolescents, and emerging adults as well as to identify any knowledge gaps.

This review followed the five stages outlined in the Arksey and O’Malley scoping review framework [11]:

### Stage 1: identifying research questions

The following question guided this scoping review: *In children, adolescents, and emerging adults, what evidence exists for ED prevention that can be delivered virtually?*

### Stage 2: identifying relevant studies

#### Eligibility criteria

Our inclusion criteria were (a) all literature, including quantitative, qualitative, and mixed methods papers on virtual prevention among children and adolescents (< 18 years) and emerging adults (18–25 years) with disordered eating symptoms/behaviours/attitudes; and (b) articles written in any language. During the screening process, the citation reviewers agreed to include studies whose participants had a mean or median age of up to and including 25.0 years.

Our exclusion criteria were (a) studies primarily involving adults (mean or median age of > 25 years); and (b) studies that included populations with clinical ED diagnoses (e.g. clinically diagnosed Anorexia Nervosa).

Virtual care is a broad term which encompasses all the methods in which healthcare providers remotely interact with their patients [15]. Prevention refers to any systematic attempt to change the circumstances that encourage, maintain, or intensify problems, where in the ED field, this includes behaviours, symptoms, and risks [16]. Prevention of EDs can occur at three levels: (1) universal (primary), where programs or interventions are aimed at entire populations, regardless of risk level; (2) selective, which targets individuals who do not yet have ED symptoms, but are at an elevated risk due to biological, psychological, or sociocultural factors; or (3) indicated (targeted), where those at high-risk due to warning signs, early symptoms and/or clear risk factors (e.g., high levels of thin-ideal internalization and body dissatisfaction), but without an ED diagnosis, are specifically targeted [16]. The focus of this review is virtual prevention at all levels, where individuals are interacting with others (or individually) remotely, aiming to reduce ED risk and symptoms. Virtual interactions can be synchronous, involving the use of audiovisual technology in real-time for communication, or asynchronous, where communication is not concurrent in time [17]. Authors of this review mutually agreed on including various synchronous and asynchronous virtual prevention modalities, including computer (e.g., CD-ROM) and internet-based (e.g., chatrooms) programs, mobile applications (‘apps’), text messaging interventions, and pre-recorded videos.

#### Databases and literature search strategy

We conducted a systematic search using the following databases: OVID Medline, PsycINFO, EMBASE, Cochrane Database of Systematic Reviews, CENTRAL, EMCARE, and CINAHL. The search included articles from 2000 to April 2021. This time frame was chosen since there was likely little or no virtual technology used in ED prevention prior to 21 years ago. We did not impose any language restrictions. The searches contained a combination of keyword and subject headings for each concept. The sample search strategy included, but was not limited to, various combinations of the following terms as appropriate for the research question: disordered eating OR body dissatisfaction OR eating concerns OR shape concerns OR weight concerns OR dietary restraint AND virtual prevention OR internet-based prevention OR computer-based prevention OR virtual self-help OR virtual psychoeducation. The references of relevant articles obtained were also reviewed. Please see Additional File 1 which contains our full search strategy.

#### Forward citation chaining

In April 2022, we used a forward citation chaining process to search each included article to see if it had been cited by any additional articles since April 2021 up until April 2022. We then screened the newly found articles to decide whether to include them. The forward chaining process involved the use of Google Scholar to locate all articles citing our included articles from the primary search.

### Stage 3: study selection

Two authors independently screened the results generated by our searches and came to consensus on which studies met eligibility criteria. We used Endnote and DistillerSR software to organize our studies. Duplicate records were removed. DistillerSR was used for article screening and data extraction. Titles and abstracts were used to exclude obviously irrelevant reports by the two reviewers. Potentially relevant articles were reviewed in full text by two reviewers who had to agree on inclusion. Articles in other languages were translated into English using Google Translate (n = 2). References of included reviews and book chapters were examined to find other potentially relevant studies. If agreement on abstract or full article inclusion could not be reached between the two reviewers, an opinion was requested from a third reviewer. There were no disputes.

### Stage 4: data charting process

A data-charting electronic form developed using DistillerSR was jointly developed by two reviewers to determine which variables to extract. Two authors independently extracted data, while continuously updating the data-charting form as needed. We extracted the following data items: general data (title, year of publication, author’s name), type of paper, methodology, mean/median age, sample size, description of virtual intervention, outcomes, and results. In line with standard scoping review practice and methodology, we did not perform a formal critical appraisal of primary studies [18, 19].

### Step 5: summarizing results

The results were organized under the following categories of prevention: asynchronous internet-based programs, synchronous internet-based programs, other e-technology (e.g., mobile apps, text messages), computer-based programs, and online caregiver prevention interventions focused on child outcomes.

We reported the review following the Preferred Reporting Items for Systematic Review and Meta-Analysis (PRISMA) guidelines-extension for scoping review [20].

## Results

### Characteristics of included studies

**Fig. 1:**
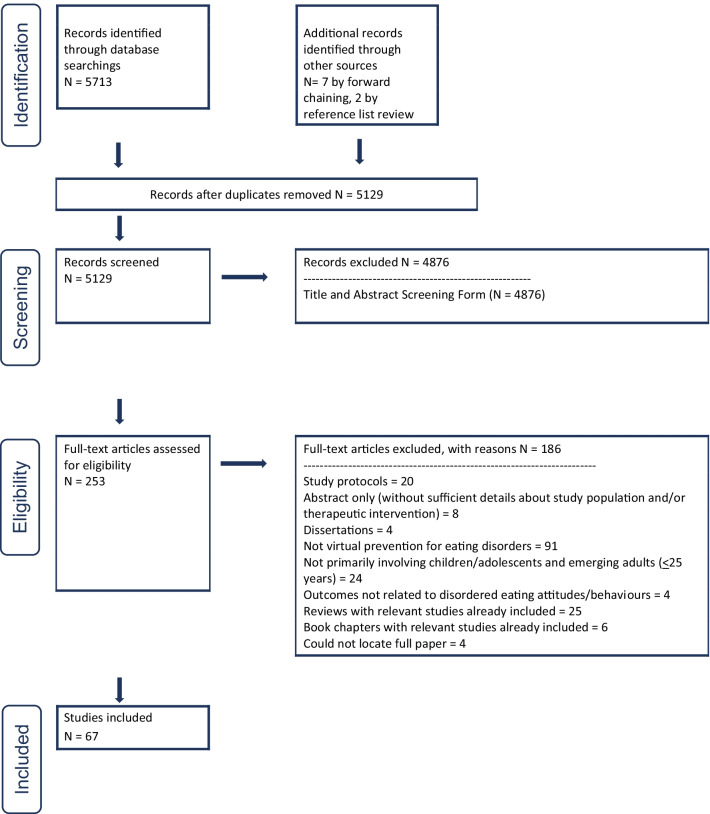
PRISMA flow diagram for virtual prevention for eating disorders for children/adolescents and emerging adults

Five thousand, seven hundred and thirteen abstracts were identified for review (see PRISMA flow diagram, Fig. 1). Seven were added with forward citation chaining up to April 23, 2022, and two more through reference list review. After duplicates were removed, abstracts were screened, where any disagreements went forward to be reviewed. Of the 253 full-text articles reviewed, 67 papers were included in our scoping review. Of these 67 papers, 58 (87%) were initially agreed upon for inclusion by the two authors at the full-text screening stage; disagreements were resolved during a consensus meeting.

### Asynchronous internet-based prevention

#### Body functionality programs

*Emerging adults* Three randomized controlled trials (RCTs) examined participants randomized to *Expand Your Horizon* (total n = 233), versus a control group (total n = 222) [21–23]. At post-test and/or 1-week follow-up, intervention participants had greater appearance satisfaction, body esteem, and body appreciation versus controls [21, 23], which were also maintained at 1-month follow-up [22]. In an adapted version of *Expand Your Horizon,* both intervention (n = 72) and active control (n = 63) participants had improvements in weight bias internalization, functionality appreciation, and self-compassion at follow-up, but effects were significantly stronger for the intervention group [24] (Table 1).

**Table 1:** Body functionality programs for emerging adults (18–25 years)

References	Type of study	Sample size	Intervention	Outcomes	Results
Alleva et al. [21]	RCT	Individuals with a desire to improve how they feel about their bodies n = 41 Expand Your Horizon n = 40 control	Expand Your Horizon: 3 online body functionality writing exercises (describing functions of the body, why they are personally meaningful, etc.); 15 min each. Active Control: creativity training program	MBSRQ-AS, BES, BAS, SOQ, OBC	At post-test and 1-week follow-up, intervention participants experienced greater appearance satisfaction, functionality (body esteem) satisfaction, and body appreciation, and lower levels of self-objectification, compared to controls. Small to medium effect sizes
Alleva et al. [22]	RCT	Individuals with a desire to improve how they feel about their bodies n = 132 Expand Your Horizon n = 129 control	Expand Your Horizon: 3 online body functionality writing exercises (describing functions of the body, why they are personally meaningful, etc.); 15 min each. Active Control: creativity training program	MBSRQ-AS, BES-physical condition subscale, BAS-2, BCQ, BSI, VAS (body satisfaction)	Relative to controls, intervention participants experienced improved appearance (body area) satisfaction, functionality satisfaction, body appreciation, and body complexity at post-test, 1-week follow-up, and 1-month follow-up. Neither body complexity nor body self-integration mediated intervention effects
Urvelyte et al. [23]	RCT	Female university students n = 60 Expand Your Horizon n = 53 control	Expand Your Horizon: 3 online body functionality writing exercises (describing functions of the body, why they are personally meaningful, etc.); 15 min each. Active Control: creativity training program	FAS, BAS-2, OBC (body surveillance subscale), BIQLI	Body functionality satisfaction and positive body image significantly increased among those in the intervention vs. control from pre- to post-intervention. The intervention was not effective in improving body image quality of life and self-objectification
Davies et al. [24]	RCT	Women with weight bias internalization (endorsed 1 item on WBIS-M) n = 72 Expand Your Horizon n = 63 Active control	Expand Your Horizon: modified version, where participants viewed 3 different videos featuring women expressing gratitude for their body functions and subsequently had to write for 10 min responding to prompts related to each video Active control: general expressive writing intervention and viewed 3 different videos, similar to the intervention	WBIS-M, FAS, SCS-SF, healthcare stress (e.g., anxiety around healthcare encounters)	While both conditions demonstrated improvements on study variables, the effects over time (from baseline to post-test and 1-week follow-up) were significantly stronger for intervention participants, including improvements in weight bias internalization, functionality appreciation, and self-compassion. Intervention participants also had significantly greater improvements in healthcare stress at follow-up compared to controls
Mulgrew et al. [25]	RCT	Female university students n = 54 Body Image program n = 63 Stress management program(control)	For both programs, 3 sessions were completed over 2 weeks (each was 30–40 min long Body Image program: Session 1-viewed videos and images related to body functionality (e.g., about diversity in women’s body shapes) and wrote reflections. Session 2–3 online writing tasks reflecting positively on body functionality, appearance, and uniqueness Stress Management program: session 1-viewed videos and images related to managing stress and maintaining a healthy headspace and wrote reflections. Session 2–3 online writing tasks on the triggers of stress, strategies to manage stress, and relaxation techniques Session 3 (for both groups): Participants had to view images representing the Western Beauty ideal and answered questions	VAS, BAS-2, BES, SOQ	Participants in both groups had improvements in body appreciation, self-objectification, and weigh concerns from pre- to post-test. Within-session state improvements were found across stress, body appreciation, appearance, and functionality satisfaction for both groups. Body Image program participants had significantly greater improvements in appearance satisfaction and body appreciation than those in the stress management program

*RCT* randomized controlled trial, *MBSRQ-AS* multidimensional body self-relations questionnaire-appearance subscales, *BES* body esteem scale, *BAS* body appreciation scale, *SOQ* self-objection questionnaire, *OBC* objectified body consciousness scale, *BCQ* body complexity questionnaire, *BSI* body-self integration scale, *VAS* visual analogue scale, *FAS* functionality appreciation scale, *BIQLI* body image quality of life inventory, *WBIS-M* modified weight bias internalization scale, *SCS-SF* self-compassion scale-short form

One RCT studied young women completing a comparable body functionality program (n = 54) versus a stress management program (n = 63), where the body functionality program participants had significantly greater improvements in their appearance satisfaction and body appreciation compared to those in the stress management program [25] (Table 1).

#### Cognitive-behavioural programs (without discussion groups)

*Children and adolescents* One mixed methods study observed usability of *Healthy Teens @ School*, among 10 students [26]. Program usability ratings increased over time, and intervention participants reported that they would recommend the program to others with mental or physical health problems (Table 2).

**Table 2:** Cognitive-behavioural programs (*without* discussion groups) for children and adolescents (< 18 years)

References	Type of study	Sample size	Intervention	Outcomes	Results
Nitsch et al. [26]	Mixed Methods (usability)	Students within normal weight range or > 85th sex age-specific BMI percentile n = 10	Healthy Teens @ School: 10-week online program (1 module per week) with 2 tracks, “Healthy Habits” and “Weight Management”	SUS, semi-structured interview (questions about impressions of program, would they recommend it, etc.)	The usability of the program was assessed in 2 rounds: the program was in the “acceptable” range in the first round, and in the “excellent” range in the second round. Participants found program content helpful and informative and would recommend it to others with psychological or health problems
Shu et al. [27]	RCT	Individuals who self-identified as having difficulties with perfectionism n = 36 ICBT-P n = 34 ICBT-S n = 24 waitlist control	ICBT-P: 8 sessions, based on CBT self-help program for perfectionism, involves learning about perfectionism and improving self-compassion ICBT-S: 8 sessions, based on a CBT self-help book for stress; education on coping styles, time management ICBT-P and ICBT-S had to complete program over 4 weeks, averaging 2 sessions per week	CPQ, EDE-Q, RCADS, RSES	ICBT-P: most favorable outcomes at 3- and/or 6-months follow-up (reducing perfectionism, ED, anxiety, depressive symptoms, increasing self-esteem). Clinical significance analysis demonstrated that ICBT-P prevented symptom increases over 6-month follow-up. ICBT-P superior to ICBT-S and controls in prevention of clinical perfectionism, depressive symptoms, and ED symptoms. No differences in attrition between the groups at 3- and 6-month follow-up. Only true preventative effects for ICBT-P vs. ICBT-S and control for depressive and ED symptoms and perfectionism, despite improvements in self-esteem (did not have significant differences in rates of deterioration among groups)

*RCT* randomized controlled trial, *SUS* system usability scale, *ICBT-P* internet cognitive behaviour therapy program for perfectionism, *ICBT-S* internet cognitive behaviour therapy for nonspecific stress management program, *CBT* cognitive behaviour therapy, *CPQ* clinical perfectionism questionnaire, *EDE-Q* eating disorder examination questionnaire, *RCADS* revised children’s anxiety and depression survey, *RSES* Rosenberg self-esteem scale, *ED* eating disorder

One RCT studied an unguided internet cognitive behaviour therapy program for perfectionism (ICBT-P; n = 36), an unguided internet cognitive behaviour therapy for nonspecific stress management program (ICBT-S; n = 34), and a waitlist control group (n = 24) [27]. Compared to ICBT-S and controls, ICBT-P resulted in the most favourable outcomes in preventing perfectionism, ED, and depressive symptoms at 3- and/or at 6-month follow-up (Table 2).

#### Cognitive-behavioural programs (with discussion groups)

*Children and adolescents* One open trial studied *Staying Fit*, where adolescents were directed to either the *Healthy Habits* track (BMI percentile < 85th for age and sex; n = 225) or *Weight Management* track (BMI percentile ≥ 85th for age and sex; n = 111) [28]. From baseline to post-intervention, BMI percentile and zBMI (standardized BMI) significantly decreased among adolescents in *Weight Management*. Individuals in *Healthy Habits* maintained their weight, and weight/shape concerns significantly decreased (Table 3).

**Table 3:** Cognitive-behavioural program (*with* discussion group) for children and adolescents (< 18 years)

References	Type of study	Sample size	Intervention	Outcomes	Results
Jones et al. [28]	Open trial	n = 336 (n = 225 “Healthy Habits” track; n = 111 “Weight Management” track)	Staying Fit: 12 online sessions (30 min each) with discussion board promoting healthy weight regulation and improved weight/shape concerns. Users directed to 1 of 2 tracks-Healthy Habits (< 85th percentile for BMI) or Weight Management (> 85th percentile for BMI). Core content adapted from Student Bodies-BED	Primary: height and weight for BMI Secondary: WCS, YRBS, CES-D, feasibility, acceptability	BMI percentile and zBMI (standardized BMI) significantly decreased among students in the Weight Management track (from baseline to post-intervention); BMI percentile and zBMI did not significantly change among those in the Healthy Habits track (maintained weight). Weight/shape concerns significantly decreased among participants in both tracks who had elevated weight/shape concerns as baseline. Students and teachers reported satisfaction with program content and implementation
Jones et al. [30]	RCT	> 85th percentile for age-adjusted BMI n = 44 SB2-BED n = 43 waitlist control	SB2-BED: 16-week online program with CBT principles from a self-help manual for BED; combines psychoeducation and behavioural exercises with monitored asynchronous discussion group	BMI, binge eating behaviour (bingeing, overeating, etc. measured with a semi-structured diagnostic interview)	Compared to controls, SB2-BED had significantly lower BMI from baseline to 9-month follow-up. Significant reductions in objective and subjective binge episodes, as well as weight and shape concerns from baseline to post-treatment and baseline to 9-month follow-up were observed among the SB2-BED group
Doyle et al. [29]	RCT	n = 40 SB2 n = 40 usual care	SB2: 16-weeks using cognitive-behavioural approach with moderated asynchronous discussion group to help overweight adolescents lose weight/improve body image. Asked to spend 1–2 h/week (max. 30 min a day) on program Usual care: received handouts containing basic information on nutrition and physical activity	BMI z-scores, EDE-Q, frequency of adolescent behavioural and cognitive skills use (e.g. self-monitoring, problem solving, seeking social support) related to eating and physical activity over past 4 months were assessed via a questionnaire, satisfaction with program	Compared to usual care, SB2 group produced a significant reduction in BMI z-scores from baseline to post intervention; SB2 group maintained reduction at 4-month follow-up, but significant differences were not observed (usual care group improved too). ED attitudes and behaviours were not significantly improved in either group at post-intervention or follow-up. SB2 group reported using healthy eating-related and physical activity-related skills more frequently vs. usual care at post-intervention and follow-up. 79% of users were satisfied with SB2; 63.2% satisfied with discussion group but 22.5% wanted more interaction
Abascal et al. [31]	RCT	General subset of sophomore students (high school) in physical education class n = 22 HRHM n = 11 HRHM-combined n = 12 other-combined n = 30 other	SB: typically, 8-weeks/sessions, but accelerated 6-week version cognitive-behavioural program with asynchronous moderated discussion group, journals, psychoeducation. Users logged in 1 day a week for 45 min; randomized to either (1) HRHM, (2) lower risk or lower motivated group, or (3) combined group. Other = lower and higher combinations (risk and motivation)	EDE-Q (eating concerns, restraint, shape concerns, weight concerns subscales), EDI, MSPSS, OSSS, WCS, motivation questionnaire, discussion group experience	HRHM group improved significantly on the EDE-Q shape concerns and weight concerns subscales from pre- to post-intervention. HRHM made significantly lower (1%) negative comments vs. HRHM-combined group (6%) in discussion groups; positive comments were also significantly higher in HRHM only group. HRHM-combined significantly improved on EDI-restraint, EDE-Q shape concerns, EDI-drive for thinness. Other-only group only significantly improved on EDE-Q shape concerns. No differences among groups on outcome measures

*Student Bodies-BED* student bodies-binge eating disorder, *BMI* body mass index, *zBMI* standardized body mass index, *WCS* weight concerns scale, *YRBS* youth risk behaviour survey, *CES-D* center for epidemiologic studies depression scale, *RCT* randomized controlled trial, *SB2-BED*-student bodies 2-binge eating disorder, *CBT* cognitive behaviour therapy, *BED* binge eating disorder, *SB2* student bodies-2, *EDE-Q* eating disorder examination questionnaire, *ED* eating disorder, *SB* student bodies, *HRHM* higher-risk higher-motivated, *EDI* eating disorder inventory, *MSPSS* multidimensional scale of perceived social support, *OSSS* online social support scale

One RCT studied *Student Bodies2* (SB2; n = 40) versus usual care (n = 40) [29] and another RCT studied *Student Bodies2-Binge Eating Disorder* (SB2-BED, specific to Binge Eating Disorder; n = 44), versus a waitlist control (n = 43) [30]. Compared to usual care, the SB2 group had significant reductions in zBMI at post-intervention, which were maintained at 4-month follow-up, but no longer significantly different from the usual care group; ED attitudes and behaviours were not significantly improved among either group at post-intervention or follow-up [29]. Compared to waitlist controls, BMI as well as binge episodes and weight/shape concerns of SB2-BED participants significantly decreased from baseline to post-treatment and 9-month follow-up [30] (Table 3).

One RCT studied an accelerated version of *Student Bodies* (SB) [31]. Students were randomized to either a higher-risk, higher-motivated SB group (HRHM-only; n = 22), lower-risk or lower-motivated SB group (Other-only; n = 30), or a SB group of HRHM and Others combined (n = 11, n = 12 respectively). From pre- to post-intervention, all groups demonstrated improvements (e.g., reduced weight and shape concerns) (Table 3).

*Emerging adults* Seven RCTs [32–38] and one cross-sectional study [39] studied *Student Bodies* (SB) among young adult females. In four RCTs, SB participants (total n = 304) demonstrated significant improvements in weight/shape concerns, from pre-post-intervention and maintained at follow-up, compared to waitlist controls (total n = 314), specifically among high-risk subgroups [32, 33, 35, 37]. Two RCTs compared SB with moderated (total n = 88) and/or unmoderated (total n = 14) discussion groups, to SB with no discussion groups (total n = 91), concluding that moderation of discussion groups may not be essential for successful outcomes [34], but that including a discussion group with SB significantly reduced weight/shape concerns from pre- to post-intervention, compared to SB without a discussion group [38]. When compared to an in-person ED psychoeducational class (n = 25), high-risk SB participants (n = 27) in one RCT significantly reduced weight/shape concerns to a greater extent at post-intervention and at 4-month follow-up [36]. A cross-section of a previous RCT [33] found increased participation in and utilization of the SB program predicted some improvements in ED behaviours [39] (Table 4).

**Table 4:** Cognitive-behavioural programs (*with* discussion group) for emerging adults (18–25 years)

References	Type of study	Sample size	Intervention	Outcomes	Results
Jacobi et al. [40]	RCT	At risk of EDs n = 51 SB + n = 52 waitlist control	SB + : 8-week/sessions cognitive-behavioural program with asynchronous moderated discussion group, journals, psychoeducation. Differs from traditional SB because adapted for subthreshold EDs by adding weekly symptom checklist and some body image exercises	EDE-Q, WCS, EDI, BSI, BDI, SCID, compliance with program	At 6-month follow-up, SB + showed significantly greater improvements in ED-related attitudes, vs. controls (medium effect sizes). SB + group showed 67% (95% CI 20–87%) greater reductions in combined rates of subjective and objective binges and 86% (95% CI = 63–95%) greater reduction in purging episodes. Rates of participants abstinent from all symptoms of disordered eating were significantly higher in SB + vs. control. Post-hoc subgroup analyses: effect on EDE-Q scores was larger/more effective in participants with binge eating than those in pure restricting subgroup
Taylor et al. [42]	RCT	High risk of EDs n = 91 intervention n = 94 waitlist control	Image and Mood: derived from SB, but 10 weekly sessions instead of usual 8 weekly sessions, cognitive behavioural program with asynchronous moderated discussion group; also differed by addressing ED risk factors	EDE, EDE-Q, SCID, BDI	ED attitudes and behaviours improved significantly more in intervention vs. control group from baseline to 2-year follow-up (moderate effect size); ED onset rate was 27% lower in intervention group vs. control (not significant). Among those with highest shape concerns, ED onset rate was significantly lower in the intervention (20%) vs. control (42%) at 2-year follow-up. Intervention might reduce ED onset for those at highest risk
Saekow et al. [43]	RCT (pilot)	High-risk for EDs n = 14 SB-ED n = 27 waitlist control (study completers)	SB-ED: 10-weekly sessions of a cognitive behavioural program with asynchronous moderated discussion group and text-based coaching (provided individualized weekly feedback)	EDE-Q, WCS, BMI, CES-D, CIA, feasibility, acceptability	SB-ED intervention had significant, medium to large effects for reduction of eating-related psychopathology, weight concerns, and psychosocial impairment, compared to waitlist controls from pre- to post-intervention. Completers rated SB-ED as very acceptable
Volker et al. [41]	RCT	At-risk of EDs (sample is same as Jacobi et al. 2012) n = 51 SB + n = 52 assessment only control	SB + : 8-weekly sessions of a cognitive-behavioural program with asynchronous moderated discussion group, journals, psychoeducation. Differs from traditional SB because adapted for subthreshold EDs, by adding weekly symptom checklist and some body image exercises	Moderators (SCID [modified], BMI, EDI-2 [drive for thinness subscale]) and Mediators (EDE-Q -shape concern subscale)	Women with higher baseline purge rates and restrictive eating might need more intensive interventions SB + effects on the reduction of binge rate were weaker for participants with higher baseline BMI and for participants with lower baseline purge rates; SB + effects on reduction of ED pathology were weaker for participants with higher baseline purge and with initial restrictive eating. No moderators of the intervention effect on restrictive eating were identified
Manwaring et al. [39]	Cross-section from Taylor et al. 2006 RCT	At-risk of EDs n = 244 consented; 209 with complete post-test data and 192 at 1-year follow-up	SB: 8-weekly/sessions of a cognitive-behavioural program with asynchronous moderated discussion group, journals, psychoeducation. Had a “booster” session available for 2 weeks 31 pprox.. 9 months following program cessation	SB utilization (e.g., number of main topic screens visited, discussion postings made or read, journal entries made, etc.), EDE-Q (restraint, eating, weight, shape concern subscales)	Total weeks participation and frequency of utilizing the online web pages/journals predicted pre- to post-treatment changes (lower scores) in EDE-Q restraint but not in other ED symptoms. Use of online discussion board was not associated with any of the outcomes from pre- to post-treatment. Treatment gains were maintained from post-treatment to 1-year follow-up. No evidence booster session was beneficial
Jacobi et al. [32]	RCT	At-risk of EDs n = 47 SB n = 50 waitlist control	SB: 8-weekly sessions of a cognitive behavioural program with asynchronous moderated discussion group, journals, psychoeducation. This version was adapted for a German population: text, culture-specific changes (e.g. German nutrition recommendations)	EDI-drive for thinness and body dissatisfaction subscales, EDE-Q, WCS, SCL-90R, BMI, SCID, compliance and general satisfaction, knowledge (e.g., information about nutrition, EDs, exercise)	SB participants maintained their improvements regarding desire to be thin and acquired knowledge about healthy eating, exercise, EDs at 3-month follow-up. Low effect sizes for total group. SB was very effective in high-risk women subgroup (n = 10 in SB, n = 12 in control), achieving significantly better changes from pre- to post-intervention and sustained at 3-month follow-up weight/shape concerns and knowledge test with larger effect sizes than for total group
Taylor et al. [33]	RCT	At-risk of EDs n = 206 SB n = 215 waitlist control	SB: 8-weekly sessions of a cognitive-behavioural program with asynchronous moderated discussion group, journals, psychoeducation	Time to onset of a subclinical/clinical ED, WCS, EDI-drive for thinness and bulimia, EDE-Q, CES-D, MSPSS, adherence	Significant reduction in weight/shape concerns in SB group vs. control at post-intervention, 1-year and 2-year follow-up. While no overall significant difference in onset of EDs between SB and controls, the SB group significantly reduced onset of EDs in 2 subgroups: participants with an elevated BMI (≥ 25) at baseline and those with baseline compensatory behaviours (vomiting, laxative, diuretic, diet pill use, driven exercise)
Low et al. [34]	RCT	At-risk of EDs n = 14 SB and moderated discussion n = 19 SB and unmoderated discussion n = 14 SB alone n = 14 controls	SB: 8-weekly sessions of a cognitive-behavioural program to address issues related to risk for EDs. Participants randomized to: SB with moderated (clinical psychologist) discussion, SB with unmoderated discussion, SB with no discussion, or control	EDI (drive for thinness, bulimia, body dissatisfaction), WCS, SATAQ (internalization and awareness), BMI, program utilization (frequency, duration of log ins)	Participation in SB resulted in better outcomes (reduced risk for eating and body image concerns) across all groups compared to controls; benefits of SB continued at 8–9-month follow-up. Participants in SB and unmoderated discussion group appeared to have the greatest reduction in ED risk. Decrease in ED risk was also associated with more time spent using the program. Moderation of discussion group may not be essential for good outcomes
Zabinski et al. [35]	RCT	At-risk of EDs n = 27 SB n = 29 control	SB: 8-weekly sessions of a cognitive-behavioural program, journals, psychoeducation; moderated discussion board was electronic bulletin board	BSQ, EDI (drive for thinness and bulimia), EDE-Q (global, restraint, eating, shape and weight concern), BMI, online social support scale, feedback about program	Both SB and controls significantly improved over time on most measures (BSQ, EDE-Q global, shape, weight). Effect sizes suggest that SB did impact the intervention group (both groups improved on BSQ but the means show that SB group reduced body image dissatisfaction to a greater extent than controls). All significant differences (except BMI) were between baseline and post-intervention and were maintained at 10- week follow-up
Celio et al. [36]	RCT	At-risk of EDs n = 27 SB n = 25 BT n = 24 waitlist control	SB: 8-weekly sessions of a cognitive-behavioural program with asynchronous moderated discussion group, journals, psychoeducation Body Traps (BT): face-to-face, psychoeducational class taught by a grad student. Met for 2 h/week over 8 weeks. Included lectures and group discussion and had same readings as SB group	EDE-Q and BSQ (weight, eating, restraint and shape concerns subscales), EDI (drive for thinness and bulimia subscales), compliance with programs	At post-treatment, SB group had significant reductions in weight/shape concerns and disordered eating attitudes vs. controls; at 4-month follow-up disordered behaviours (body image dissatisfaction) were also reduced (strongest results at follow-up). No significant effects were found between controls and BT group. Compliance was better with SB than BT. Among high-risk participants, SB was significantly more effective than BT and controls in reducing weight/shape concern at post-treatment and 4-month follow-up
Winzelberg et al. [37]	RCT	At-risk of EDs n = 24 SB n = 20 control	SB: 8-weekly sessions of a cognitive-behavioural program with asynchronous moderated discussion group, journals, psychoeducation	BSQ, EDI (drive for thinness and bulimia), EDE-Q (weight concerns and shape concerns), OSSS, compliance to program, frequency and theme of discussion group postings	No significant differences between SB and control at post-intervention, but at 3-month follow-up significant differences were found between BSQ and EDI drive for thinness (favouring SB). Weekly compliance decreased during the intervention. Compliance significantly related to improvement on BSQ. High level of participation in discussion group, but participants reported receiving only a moderate level of social support from the group
Kass et al. [38]	RCT	At-risk of EDs n = 74 SB and moderated discussion group n = 77 SB and no discussion group	SB: 8-weekly sessions of a cognitive-behavioural program with asynchronous moderated discussion group, journals, psychoeducation The comparator group received SB but without the discussion group component	WCS, EDE-Q, BDI-II, MES, body composition (BMI), adherence to program	Weight/shape concerns were reduced more significantly among SB group with guided discussion group than SB with no discussion group. Guided discussion group participants had 67% lower odds of having high-risk weight/shape concerns post-intervention. Those who logged into the program in the guided discussion group spent significantly more time using the program than did those in the no discussion group condition
Ohlmer et al. [44]	Open trial (pilot)	At-risk for AN specifically n = 36	SB-AN: cognitive-behavioural prevention program for AN, modelled after SB + but expanded to 10 weekly sessions (instead of 8), each 45–90 min. Also added motivational interviewing elements, more psychoeducation on EDs, focus on restrictive eating; included online discussion group, weekly feedback, weekly symptom checklist	Feasibility, adherence, and acceptance; WCS, EDE-Q, EDI-2, BSI, BDI, FMPS, knowledge test	32 women at post-intervention and 26 at 6-month follow-up. High satisfaction with the program. Significant and stable improvements with medium to large effect sizes on most variables of disturbed eating (WCS, EDE-Q, EDI-2 drive for thinness) at post-intervention and 6-month follow-up. BDI improved significantly at 6-month follow-up. EDI-2 bulimia and body dissatisfaction and associated psychopathology showed inconsistent improvements with medium to small effect sizes. Low-weight and normal-weight group: effects were mostly comparable with overall effects; underweight group, significant increase in BMI at post and follow-up; binge eating group: total number of binges in past 28 days was lower at follow-up vs pre-intervention
Melioli et al. [45]	Meta-analysis	n = 20 studies	Student Bodies, My Body My Life, Student Bodies2, Student Bodies + , other internet-based prevention programs For this meta-analysis: between-group effect sizes were calculated for ED symptoms and risk factors	BSQ, EDI (drive for thinness, bulimia, body dissatisfaction), EDE-Q, WCS, CES-D, SATAQ-3, PACS, BULIT-R, DEBQ-R, EWLB, BDI-SF, BDI-II, EAT-40, BITE	Compared with controls, internet-based programs significantly decreased body dissatisfaction, thin- ideal internalization, shape and weight concern, dietary restriction, drive for thinness, bulimic symptoms, purging frequency, and negative affect (small to moderate effect sizes). No evidence of negative effects of internet-based prevention programs
Beintner et al. [46]	Meta-analysis	n = 10 studies (6 US and 4 Germany) n = 990 (504 SB, 486 waitlist control)	SB: 8-weekly sessions of a cognitive-behavioural program with asynchronous moderated discussion group, journals, psychoeducation	WCS, BSQ, EDI/EDI-2-drive for thinness, bulimia and body dissatisfaction, EDE-Q- restraint, eating concern, weight concern, shape concern	At post-intervention, moderate effect sizes were found across all studies for EDI-drive for thinness, WCS, BSQ; small effect sizes for EDI-bulimia and EDE-Q-restraint, eating concern, weight concern, shape concern. At follow-up (12 weeks-12 months), a moderate effect size was found for EDI-drive for thinness and body dissatisfaction subscales, WCS, BSQ, EDE-Q restraint, shape concern subscales. Effect sizes for EDI-bulimia and EDE-Q eating concern and weight concern subscales were small. SB associated with mild to moderate improvements in ED attitudes (especially reductions of negative body image and desire to be thin); reported effects generally maintained
Beitner et al. [47]	Meta-analysis	n = 10 studies (6 United States, 4 Germany) n = 990 (504 SB, 486 waitlist control)	SB: 8-weekly sessions of a cognitive-behavioural program with asynchronous moderated discussion group, journals, psychoeducation	Participant adherence, EDI-drive for thinness and bulimia	Adherence predicted intervention effects on the EDI Drive for Thinness, but not on the EDI Bulimia subscale. Adherence to SB proved to be high across a number of trials, settings, and countries (Germany and United States)

*RCT* randomized controlled trial, *ED* eating disorder, *SB* + student bodies + , *EDE-Q* eating disorder examination questionnaire, *WCS* weight concerns scale, *EDI* eating disorder inventory, *BSI* brief symptom inventory, *BDI* Beck depression inventory, *SCID* structured clinical interview for DSM disorders, *CI* confidence interval, *SB* student bodies, *EDE* eating disorder examination, *SB-ED* student bodies-eating disorders, *BMI* body mass index, *CES-D* center for epidemiologic studies depression scale, *CIA* clinical impairment assessment, *SCL-90* symptom checklist-90, *MSPSS* multidimensional scale of perceived social support, *SATAQ* sociocultural attitudes towards appearance questionnaire, *BSQ* body shape questionnaire, *BT* body traps, *OSSS* online social support scale, *BDI-II* Beck depression inventory-2, *MES* motivation and expectation scale, *AN* anorexia nervosa, *SB-AN* student bodies-anorexia nervosa, *FMPS* frost multidimensional perfectionism scale, *PACS* physical appearance comparison scale, *BULIT-R* bulimia test-revised, *DEBQ-R* Dutch eating behaviour questionnaire-restraint, *EWLB* extreme weight loss behaviour scale, *BDI-SF* Beck depression inventory-short form, *EAT-40* eating attitudes test, *BITE* bulimic investigatory test Edinburgh

One RCT [40] determined that SB + (variation of SB) participants (n = 51) significantly improved in ED-related attitudes versus waitlist controls (n = 52) at 6-month follow-up, however a subsequent study [41] found SB + effects on ED pathology were weaker for participants with higher baseline purging and restrictive eating. One RCT studied another adapted version of SB (*Image and Mood;* n = 91), versus waitlist controls (n = 94), and among those with the highest shape concerns at baseline, ED onset rate was significantly lower in the intervention group (20%) compared to controls (42%) at 2-year follow-up [42]. One pilot RCT studied *Student Bodies-Eating Disorders* (SB-ED), and found SB-ED (n = 14) significantly reduced eating-related psychopathology, weight concerns, and psychosocial impairment, versus waitlist controls (n = 27) from pre- to post-intervention [43]. In a pilot open trial testing *Student Bodies-Anorexia Nervosa* (SB-AN), intervention completers demonstrated significant improvements in disturbed eating attitudes and behaviours at post-intervention (n = 32) and maintained at 6-month follow-up (n = 26) [44] (Table 4).

Three meta-analyses reported similar results of SB and its adapted versions, highlighting the effect of the interventions in significantly decreasing body dissatisfaction, thin-ideal internalization, ED symptoms, and weight/shape concerns at post-intervention and various follow-up times [45, 46], as well as demonstrating high adherence to SB interventions across different trials, settings, and countries [47] (Table 4).

#### Cognitive-restructuring and gratitude intervention

*Emerging adults* One RCT compared three e-workbook conditions: a gratitude intervention (n = 35), a cognitive restructuring intervention (n = 28), and a control group (n = 45) [48]. The gratitude intervention outperformed the other conditions, evident through improvements such as increased body esteem and decreased body dissatisfaction from pre- to post-intervention; the cognitive restructuring intervention did not seem to provide much benefit (Table 5).

**Table 5:** Cognitive-restructuring and gratitude intervention for emerging adults (18–25 years)

References	Type of study	Sample size	Intervention	Outcomes	Results
Wolfe and Patterson [48]	RCT	General female undergraduate students n = 35 Gratitude intervention n = 28 Cognitive Restructuring intervention n = 45 Control	Gratitude: emailed a workbook with instructions to create a gratitude list every day for 14 days Cognitive Restructuring: emailed a workbook with instruction to complete automatic thought records every day for 14 days Control: emailed brief educational workbook on body image	CES-D, PANAS, BINGE, EAT-26, BSQ, BES, BAS	Pre- to post-intervention: gratitude intervention outperformed other conditions at increasing body esteem, decreasing body dissatisfaction, reducing dysfunctional eating, and reducing depressive symptoms. Cognitive restructuring intervention had a significant increase in depressive symptoms and a significant decrease in positive mood (from pre- to post-intervention). A significantly greater proportion of cognitive restructuring participants dropped out compared to gratitude participants

*RCT* randomized controlled trial, *CES-D* center for epidemiologic studies depression scale, *PANAS* positive and negative affect scale, *BINGE* binge eating scale, *EAT-26* eating attitudes test 26 items, *BSQ* body shape questionnaire, *BES* body esteem scale, *BAS* body appreciation scale

#### Imagery rescripting program

*Emerging adults* One RCT studied Body Image Rescripting (BIR; n = 28), General Image Rescripting (GIR; n = 31), psychoeducation (n = 34) and a control group (n = 25) [49]. All three active interventions had a significant impact on reducing global eating pathology and increasing body acceptance. Compared to controls, BIR participants improved in self-compassion, while those in the GIR group improved dysfunctional attitudes such as clinical perfectionism, versus controls (Table 6).

**Table 6:** Imagery rescripting program for emerging adults (18–25 years)

References	Type of Study	Sample Size	Intervention	Outcomes	Results
Zhou et al. [49]	RCT	General female university students n = 28 BIR n = 31 GIR n = 34 Psychoeducation n = 25 Control	BIR and GIR: asked to write about an earlier memory, in first and third person; to practice imagery rescripting for 5 min/day for a week Psychoeducation: received a handout (how EDs affect brain) and 10 min learning quiz Control: asked to let their mind wander for 10 min after negative mood induction	WCS, EDE-Q, BI-AAQ, SCS-SF, FSC, PANAS, BISS	BIR, GIR, and psychoeducation had significant impact on global eating psychopathology and body acceptance. BIR improved self-compassion and fear of self-compassion (e.g., fears of unworthiness, becoming ‘weak’) and GIR improved dysfunctional attitudes (clinical perfectionism and low self-esteem) compared to control. Additionally, BIR was found to improve self-compassion only compared to psychoeducation (but not control)
Pennesi and Wade [50]	RCT	Female university students at-risk of EDs n = 37 Imagery rescripting n = 35 Cognitive dissonance n = 35 Control	Imagery Rescripting: wrote a past memory of an unpleasant body experience and how their body looked from an observer’s perspective and in the present with self-compassion Cognitive Dissonance: read a definition of the thin-ideal stereotype for women and viewed accompanying images; brainstormed consequences of pursuing this ideal and positive attributes about themselves Control: Participants asked to let their mind wander	EDE-Q, CIA, SCS-SF, DASS-21, BI-AAQ	Findings provide some qualified support for the imagery rescripting techniques over the dissonance-based techniques and control conditions. Imagery rescripting was associated with significant improvements in body image acceptance compared to the cognitive dissonance condition, but not compared to the control condition. Imagery rescripting was associated with significant improvements in self-compassion and levels of disordered eating compared to the control condition but not compared to the cognitive dissonance condition

*RCT* randomized controlled trial, *BIR* body image rescripting, *GIR* general image rescripting, *ED* eating disorder, *WCS* weight concerns scale, *EDE-Q* eating disorder questionnaire, *BI-AAQ* body image acceptance and action questionnaire, *SCS* self-compassion scale, *FCS* fear of self-compassion scale, *PANAS* positive and negative affect schedule, *BISS* body image state scales, *CIA* clinical impairment assessment questionnaire, *DASS-21* depression, anxiety, and stress scale-short form (21 items)

Similarly, another RCT studied imagery rescripting (n = 37), cognitive dissonance (n = 35), and a control group (n = 35) [50]. Those in the imagery rescripting condition had significant improvements in body image acceptance compared to the cognitive dissonance group, and significant improvements in self-compassion and levels of disordered eating compared to control group (Table 6).

#### Media literacy programs

*Emerging adults* Two RCTs studied *Media Smart-Targeted* (MS-T). One RCT compared MS-T (n = 122) to a control group (n = 194), and found MS-T participants were 66% less likely than controls to develop an ED by 12-month follow-up (non-significant) [51]. Another RCT compared the same intervention and control groups, to a group completing SB (n = 98) [52]. MS-T participants had significant improvements in depression (at 6- and 12-month follow-up), internalization (post-intervention), and clinical impairment [12-month follow-up] versus SB participants (Table 7).

**Table 7:** Media literacy (targets media internalization) programs for emerging adults (18–25 years)

References	Type of study	Sample size	Intervention	Outcomes	Results
Wilksch et al. [51]	RCT	Females seeking to improve body image n = 122 MS-T n = 194 Control	MS-T: a 9-module program released weekly; online adaptation of Media Smart, a school-based program that has been found to reduce ED risk. MS-T has a greater focus on social media pressures, emotion regulation, goal setting, addressing eating-related risk factors. Control: received tips for positive body image	EDE-Q	220 participants (69.6%) met criteria for ED at baseline but lacked a formal diagnosis. MS-T participants were 66% less likely than controls to develop an ED by 12-month follow-up (nonsignificant). MS-T participants who met ED criteria at baseline were 75% less likely than controls to still meet diagnostic criteria at follow-up. This effect was significant and remained so for both those who did and who did not access external face-to-face ED treatment during the trial
Wilksch et al. [52]	RCT	Females seeking to improve body image ITT analyses: n = 122 MS-T n = 98 SB n = 194 Control Measure completer analyses: n = 82 MS-T n = 70 SB n = 169 Control	MS-T and SB: 9-module programs including interactive content to target ED risk factors. Both released modules weekly via a password-protected mobile internet-platform and were pure self-help format (no online therapist moderator). SB focused on risk factors for disordered eating while MS-T focused more on media internalization. Control: emailed 10 tips on positive body image	EDE-Q, WCS, DASS-21, EDI, CIA, SATAQ-3	Primary ITT analyses revealed no differences between groups; measure completer analyses found MS-T had significantly lower EDE-Q Global than controls at 12-month follow-up. Secondary ITT analyses found MS-T participants reported significantly higher quality of life–mental relative to SB and controls (6-month follow-up). Of those with baseline disordered eating, MS-T participants were significantly less likely than controls to report it at 12-month follow-up. Small to medium effect sizes between MS-T and SB (favouring MS-T; significant) on depression, (6-and 12-month follow-up), internalization (post-program), clinical impairment (12-month follow-up), etc

*RCT* randomized controlled trial, *MS-T* media smart-targeted, *ED* eating disorder, *EDE-Q* eating disorder examination questionnaire, *SB* student bodies, *WCS* weight concerns scale, *DASS-21* depression, anxiety and stress scale-short form (21 items), *EDI* eating disorder inventory, *CIA* cinical impairment assessment questionnaire, *SATAQ-3* sociocultural attitudes towards appearance questionnaire-3, *ITT* intention to treat

#### Self-compassion exercises

*Emerging adults* One RCT studied undergraduate women completing a self-compassion meditation podcast intervention (n = 40) or a waitlist control (n = 40) [53]. Compared to controls, there were significantly greater pre-post changes among the intervention group on body appearance, body surveillance, and appearance contingent-self-worth, however no significant improvements in total self-compassion or body shame/dissatisfaction were observed. A different RCT studied female college students randomized to self-compassion (n = 51), traditional expressive (n = 50) or control (n = 51) online writing exercises [54]. Contrary to the RCT described previously [53], there were significantly greater increases in self-compassion among the self-compassion writing group compared to the traditional expressive writing and control groups (Table 8).

**Table 8:** Self-compassion exercises for emerging adults (18–25)

References	Type of study	Sample size	Intervention	Outcomes	Results
Toole and Craighead [53]	RCT	Undergraduate women endorsing body image or appearance concerns n = 40 Intervention n = 40 Waitlist control	Self-compassion meditation training: first received training in-person in a lab, then were emailed a link each day to a self-compassion meditation training podcast; asked to listen to podcasts (20 min each) daily for 1 week in a private space, ideally at the same time each day, and at a time when they felt alert	SCS, BAS, RSES, Body Surveillance and Body Shame subscales of OBCS, BSQ, CSW-Appearance, practice frequency, acceptability	No significant improvements in total self-compassion scores or body shame and body dissatisfaction after intervention, but intervention groups showed significantly greater pre-post changes on body appearance, appearance contingent self-worth scales, and body surveillance vs controls. Results suggest that brief exposure to self-compassion holds promise for improving aspects of self-compassion and body image distress; has the potential to be an acceptable and cost-effective method to reduce body image distress
Zeimer et al. [54]	RCT	Female undergraduate college students n = 51 self-compassion writing n = 50 traditional expressive writing n = 51control	All groups had to write for 20 min once/week (online) for 3 consecutive weeks Self-Compassion Writing: participants had to write about their body image and related experiences from a self-compassionate perspective (e.g., expressed understanding, kindness, and concern to themselves in a manner that a concerned friend may respond) Traditional Expressive Writing: participants had to write about their deepest feelings associated with their body image Control: participants had to describe the events of their day in a factual and detail-oriented way, focusing on information only rather than thoughts or feelings about the day’s events	BAS-2, Body Image Quality of Life Inventory, PANAS, 26-item Self-Compassion Scale	The self-compassion group experienced significantly greater increases in self-compassion (medium effect size) vs. traditional expressive and control groups; body appreciation and body image quality of life was mediated by self-compassion. Group differences were not significant for any other outcome variables (e.g., there were no differences between groups on positive body image or affect. All 3 groups had significant decreases in positive affect (small effect) and negative affect (medium effect)

*RCT* randomized controlled trial, *SCS* self-compassion scale, *BAS* body appreciation scale, *RSES* Rosenberg self-esteem scale, *OBCS* objectified body consciousness scale, *BSQ* body shape questionnaire, *CSW-Appearance* contingencies of self-worth scale-appearance subscale, *PANAS* positive and negative affect schedule

#### Psychoeducation programs

*Children and adolescents* One RCT studied high school males (n = 39) and females (n = 86) randomized to *BodiMojo*, (website version) or a control [55]. The intervention decreased body dissatisfaction and increased appearance esteem in females, but this was not maintained at 3-month follow-up. Compared to females, males were not as engaged in the intervention (Table 9).

**Table 9:** Psychoeducation programs for children and adolescents (< 18)

References	Type of study	Sample size	Intervention	Outcomes	Results
Franko et al. [55]	RCT	High school students n = 39 boys n = 86 girls	BodiMojo: website aiming to promote health behavior change through technology and social engagement; provides relevant information and feedback, goal setting, body image content, interactive games, quizzes, videos that are specific to this adolescent age group; students used in 45-min class periods over 4 weeks. Controls: continued attending standard health education courses in classrooms	Satisfaction survey regarding intervention, PACS, EDI-body dissatisfaction scale, BES	BodiMojo decreased body dissatisfaction and increased appearance satisfaction and body esteem among girls, although the comparative effects were modest and not maintained at follow up. BodiMojo was successful in decreasing appearance comparison. Girls with high initial levels of body dissatisfaction, as well as those who were overweight, reported the greatest improvements in body dissatisfaction. Boys were not as engaged

*RCT* randomized controlled trial, *PACS* physical appearance comparison scale, *EDI* eating disorder inventory, *BES* body esteem scale

### Synchronous internet-based prevention

#### Internet-based chatrooms

*Children and adolescents* One RCT compared *My Body, My Life: Body Image Program for Adolescent Girls* intervention (n = 28) to a delayed treatment control (n = 34) [56]. Among intervention completers, clinically significant improvements in body dissatisfaction, disordered eating, and depression were observed at post-intervention and maintained at 2- and 6-month follow-up. Internet-delivery was also enthusiastically endorsed (Table 10).

**Table 10:** Internet-based chatrooms (*with synchronous* discussion groups) for children and adolescents (< 18 years)

Reference	Type of Study	Sample Size	Intervention	Outcomes	Results
Heinicke et al. [56]	RCT	Adolescent females self-identifying as having body image or eating problems n = 28 intervention n = 34 delayed treatment control	My Body, My Life: Body Image Program for Adolescent Girls: 6, 90-min weekly small group (4–8) synchronous sessions led by a therapist and manual based on cognitive-behavioural principles and psychoeducation; conducted online in a secured chatroom	BSQ-short form, BMI, BCS, DEBQ-R, EWLB, EDI-B, SATAQ-3, BDI-SF, qualitative evaluation (assessing thoughts on internet delivery mode, practicality, etc.)	Among intervention completers, clinically significant improvements in body dissatisfaction (BSQ-SF), disordered eating (DEBQ-R), and depression (BDI-SF) were observed at post-intervention and maintained at follow-up (2 and 6 months). Internet delivery was enthusiastically endorsed: 65% reported preferring to participate in a program delivered via internet; 15% would prefer face-to-face. 42.3% felt comfortable sharing with a group; 77% felt program markedly/moderately improved their body image

*RCT* randomized controlled trial, *BSQ* body shape questionnaire, *BMI* body mass index, *BCS* body comparison scale, *DEBQ-R* Dutch eating behaviour questionnaire-restraint, *EWLB* extreme weight loss behaviours scale, *EDI-B* eating disorder inventory-bulimia subscale, *SATAQ-3* sociocultural attitudes towards appearance scale-3, *BDI-SF* Beck depression inventory-short form

*Emerging adults* One pilot open trial found significant improvements in ED symptoms at post-intervention and 10-week follow-up, following a 7-week moderated, online chatroom program [57]. In an RCT testing the same chatroom program (but 8-weeks in duration), participants were randomized to either the chatroom (n = 28), or a waitlist control (n = 30) [58]. Like the open trial, chatroom participants showed significantly reduced body shape and eating concerns and improved self-esteem over controls at post-treatment and 10-week follow-up. In both studies, participants reported a preference for online instead of face-to-face discussions surrounding their ED-related issues (Table 11).

**Table 11:** Internet-based chatrooms (*with synchronous* discussion group) for emerging adults (18–25 years)

References	Type of study	Sample size	Intervention	Outcomes	Results
Zabinski et al. [58]	RCT	Undergraduate females who scored > 57 on WCS, at risk of ED n = 28 intervention chatroom n = 30 waitlist control	Internet chatroom: once per week for 8 weeks, used a private chatroom for a 1-h moderated discussion focused on improving body image and eating behaviours; also included psychoeducation, asynchronous support, homework	EDE-Q (eating concern, restraint, shape concern and weight concern), RSES, MSPSS, OSSS, satisfaction questionnaire	Intervention participants significantly reduced eating pathology (EDE-Q shape concern, eating concern) and improved self-esteem over controls at post-treatment, and 10-week follow-up. High satisfaction with intervention mode (86% satisfied or very satisfied with program; 79% felt chats were better or much better than face-to-face)
Zabinski et al. [57]	Open trial (pilot)	Undergraduate females with high BSQ scores n = 4	Moderated, synchronous Internet Relay Chat (IRC): 7-week online chatroom program. Had to complete weekly readings, chat discussions, and summaries of each chat. Chatroom component was for 1 h/week. Content of readings/homework was based on cognitive-behavioural approach to address unhealthy eating practices and negative body image/attitudes	BSQ, EDI (drive for thinness and bulimia subscales), EDE-Q, RSES, OSSS, acceptability of program	Participants rated satisfaction with the program very highly; believed it was easier for them to be honest on the computer vs. face-to-face discussion. All reported that the program helped to prevent negative attitudes about their weight and shape from making them feel badly, and to recognize the thoughts and situations that trigger negative feelings/behaviours. All reported a positive overall experience with the group. Almost all ED measures showed improvement at post-intervention and 10-week follow-up except for EDI-Bulimia (skewed by 1 participant). No changes on RSE. BMI remained stable from pre to post

*RCT* randomized controlled trial, *WCS* weight concerns scale, *ED* eating disorder, *EDE-Q* eating disorder examination questionnaire, *RSES* Rosenberg self-esteem scale, *MSPSS* multidimensional scale of perceived social support, *OSSS* online social support scale, *BSQ* body shape questionnaire, *EDI* eating disorder inventory

#### Dissonance-based programs

*Children and adolescents* One RCT studied *virtual Body Project* (vBP; n = 149), an expressive writing condition (EW; n = 148), and a control group (n = 149) [59]. Participants in vBP had a significantly greater reduction in ED symptoms, body dissatisfaction, and internalization of thin-ideal versus EW at post-intervention, and up to 24-months follow-up (Table 12). In another RCT, participants were randomized *to eBody Project* (eBP; adapted version; n = 21), or a control group (n = 128) [60], where eBP participants reported significant improvements in body dissatisfaction and restrained eating, compared to controls at 6-month follow-up (Table 12).

**Table 12:** Dissonance-based programs for children and adolescents (< 18 years old)

References	Type of study	Sample size	Intervention	Outcomes	Results
Ghaderi et al. [59]	RCT	Females with body image concerns n = 149 vBP n = 148 EW n = 149 Control	vBP: 4 weekly sessions (1 h each), critiquing the thin-ideal delivered via Google hangouts for 4 weeks EW: Instructions sent weekly for 4 weeks where participants wrote about thoughts and emotions about their body for 40 min	EDE, EDDS, PANAS, EDE-Q (restraint subscale), CIA, BPDS, BSQ, IBSS-R	Incidence of ED onset in vBP participants was 77% less than in EW participants by 24 month follow up. vBP participants generally showed significantly greater reduction in ED symptoms, clinical impairment, body dissatisfaction, internalization of thin-ideal compared with the waitlist participants at postintervention and 6-month follow-up, and in ED symptoms, restraint, body dissatisfaction, and internalization of thin-ideal compared with the EW participants at postintervention, and 6-, 12-, 18-, or 24-months follow-up
Luo et al. [60]	RCT	Females from China with body dissatisfaction n = 21 eBody Project n = 128 Control	eBody Project (adapted Chinese version used): 6 internet modules over 6 weeks (35–45 min each) critiquing feminine attractiveness ideal; aiming to increase self-acceptance and to induce dissonance about pursuing a thin-ideal Education Brochure Control Intervention: 2 pages, content included descriptions of positive and negative body image, key consequences of negative body image, particularly regarding increased risk for ED onset; had to review it for 35–45 min/week for 6 weeks	BDS, IBSS-R, CES-D, RSES, BAS-2, EDDS, DRES	eBody Project women reported significant decreases in body dissatisfaction from baseline to post-treatment and baseline to 6-month follow-up; no significant changes in controls. Self-esteem significantly improved for eBody project women from baseline to post treatment and remained stable at follow up (brochure had no significant changes). eBody Project women showed significant decreases in restrained eating vs. controls in assessments of changes from baseline to post-treatment and follow-up. eBody Project women showed significantly sharper decreases in ED symptoms in the baseline to follow-up evaluation than controls. Corresponding effect sizes were small to medium

*RCT* randomized controlled trial, *vBP* virtual body project, *EW* expressive writing, *EDE* eating disorder examination, *EDDS* eating disorder diagnostic scale, *PANAS* positive and negative affect schedule, *EDE-Q* eating disorder examination questionnaire, *CIA* clinical impairment assessment, *BPDS* body parts dissatisfaction scale, *BSQ* body shape questionnaire, *IBSS-R* ideal body stereotype scale revised, *ED* eating disorder, *BDS* body dissatisfaction scale, *CES-D* center for epidemiologic studies depression scale, *RSES* Rosenberg self-esteem scale, *BAS-2* body appreciation scale-2, *DRES* Dutch restrained eating scale

*Emerging adults* Of all synchronous internet-based prevention interventions, the eBP program was the most studied amongst young adult women, although findings were mixed. Two RCTs studied facilitator-led, in-person *Body Project* (BP; total n = 39), eBP (internet-based; total n = 19), educational video control (total n = 29), or educational brochure control (total n = 20) [61, 62]. BP and eBP participants similarly showed greater pre-post reductions in some ED risk factors (e.g., body dissatisfaction and self-reported dieting) than both controls [61]; longer term follow-up suggests that effects faded more quickly for eBP than BP [62]. Three larger RCTs and one qualitative study investigated clinician-led BP (total n = 173), peer-led BP (total n = 162), eBP (total n = 184), or educational video control (total n = 161) [63–66]. One of these studies found significant reductions in ED risk factors (thin-ideal internalization, body dissatisfaction, negative affect), and ED symptoms in eBP participants at post-test versus controls, however, clinician- and peer-led BP groups showed significantly greater effects than eBP [65]. In a follow-up study, clinician-, peer-led, and eBP groups had larger reductions in ED risk factors and symptoms versus controls at various follow-up periods, but peer-led groups had greater improvements in some ED risk factors (e.g. body dissatisfaction) compared to eBP [63]. Another study indicated that age moderated intervention effects, such that in-person BP was superior to eBP in reducing ED symptomatology in women up to 20 years old [64]. Additionally, qualitative reports indicate that some eBP participants felt more support was needed to improve experiences in the program [66] (Table 13).

**Table 13:** Dissonance based programs for emerging adults (ages 18–25)

References	Type of study	Sample size	Intervention	Outcomes	Results
Stice et al. [63]	RCT	Females with high body image and/or body dissatisfaction concerns n = 173 Clinician-led Body Project n = 162 Peer-led Body Project n = 184 eBody Project n = 161 Control	Clinician-led Body Project: in-person group and led by clinician Peer-led Body Project: in -person group and led by peers eBody Project: six 40-min modules (overall same timing as clinician- and peer-led groups) involving user-driven educational activities and games (e.g., texting role-plays) that parallel the in-person group program (e.g. in group, engaged in exercises critiquing thin beauty ideal) Educational Video Control: watched 55 min video “Dying to be Thin”	Thin-Ideal Internalization scale, Satisfaction and Dissatisfaction with Body Parts Scale, DRES (frequency of dieting), PANAS-X, EDDI	Participants in clinician- and peer-led Body Project groups and the eBody Project generally showed larger reductions in risk factors and ED symptoms vs controls through 1- and 2-year follow-up, with some effects persisting through 3- and 4-year follow-ups (no other Internet-delivered prevention program has reduced ED symptoms, produced greater reductions in outcomes than an alternative educational intervention, or produced effects that have persisted through 4-year follow-up). Peer-led Body Project participants showed greater reductions in some ED risk factors than eBody Project participants. Might be optimal to task-shift Body Project delivery to peer-leaders
Rohde et al. [64]	RCT	Females with high body image and/or body dissatisfaction concerns n = 173 Clinician-led Body Project n = 162 Peer-led Body Project n = 184 eBody Project n = 161 Control	Clinician-led Body Project: in-person group and led by clinician Peer-led Body Project: in -person group and led by peers eBody Project: six 40-min modules (overall same timing as clinician- and peer-led groups) involving user-driven educational activities and games (e.g., texting role-plays) that parallel the in-person group program (e.g., in group, engaged in exercises critiquing thin beauty ideal) Educational Video Control: watched 55 min video “Dying to be Thin”	Thin-Ideal Internalization Scale, Satisfaction and Dissatisfaction with Body Parts Scale, DRES (frequency of dieting), EDDI, SAS	Attrition: 10% at post-test and 16% at follow-up. Interactions indicated that age moderated the intervention effects, such that group- based programs were superior to the Internet-delivered version in terms of ED symptom reductions for women up to age 20
Stice et al. [65]	RCT	Females with high body image and/or body dissatisfaction concerns n = 173 (clinician-led) n = 162 (peer-led) n = 184 (eBody Project) n = 161 (control)	Clinician-led Body Project: in-person group and led by clinician Peer-led Body Project: in -person group and led by peers eBody Project: six 40-min modules (overall same timing as clinician- and peer-led groups) involving user-driven educational activities and games (e.g., texting role-plays) that parallel the in-person group program (e.g. in group, engaged in exercises critiquing thin beauty ideal) Educational Video Control: watched 55 min video “Dying to be Thin”	Thin-Ideal Internalization Scale Satisfaction and Dissatisfaction with Body Parts Scale sadness, guilt, and fear/anxiety subscales from the PANAS-X, semi-structured EDDI	Attrition 9% at posttest and 11% at 6-month-follow up. eBody Project participants showed significantly greater reductions in all 4 continuous outcomes (thin-ideal internalization, body dissatisfaction, negative affect, ED symptoms) by posttest compared to controls. The effects for thin-ideal internalization and ED symptoms were still significant by 6- month follow-up compared to controls. Participants in clinician-led and peer-led Body Project groups showed significantly greater reductions in ED risk factors than eBody Project participants
Chithambo and Huey [67]	RCT	Females at high-risk for EDs n = 90 DBI-I n = 88 CBI-I n = 93 No intervention	DBI-I: 4 internet-delivered weekly sessions with activities to argue against thin-ideal (content from Body Project) CBI-I: 4 internet-delivered weekly sessions based on model positing that negative body image thoughts and assumptions sustain disturbed body evaluation and maladaptive eating (content from The Body Image Workbook, a self-help manual for body dissatisfaction)	BSQ, IBSS, EAT, DRES, BDI-II	At postintervention, DBI-I and CBI-I led to greater reductions in body dissatisfaction, thin-ideal internalization, and depression than no intervention (no differences between CBI-I and DBI-I). CBI-I was effective at reducing dieting and composite eating pathology relative to no intervention
Haderlein et al. [68]	Secondary analysis of Chithambo & Huey 2017 RCT	Females at high-risk for EDs (≥ 34 WCS score) n = 88 DBI-I n = 94 CBI-I n = 96 no intervention	DBI-I: 4 internet-delivered weekly sessions with activities to argue against thin-ideal (content from Body Project) CBI-I: 4 internet-delivered weekly sessions based on model positing that negative body image thoughts and assumptions sustain disturbed body evaluation and maladaptive eating (content from The Body Image Workbook, a self-help manual for body dissatisfaction)	RED scale	DBI-I was associated with significantly greater reductions in reward-based eating drive (RED scores) over time vs. the no intervention group. CBT-I had no effect on reward-based eating drive. There was no time x condition effect between DBI-I and CBT-I, nor for CBT-I and no intervention
Shaw et al. [66]	Qualitative Study	Females with high body image and/or body dissatisfaction concerns n = 173 Clinician-led Body Project n = 162 Peer-led Body Project n = 184 eBody Project n = 161 Control	Clinician-led Body Project: in-person group and led by clinician Peer-led Body Project: in -person group and led by peers eBody Project: six 40-min modules (overall same timing as clinician- and peer-led groups) involving user-driven educational activities and games (e.g., texting role-plays) that parallel the in-person group program (e.g., in group, engaged in exercises critiquing thin beauty ideal) Educational Video Control: watched 55 min video “Dying to be Thin”	Participants completed exit surveys with 4 open ended questions addressing: valuable and less valuable aspects of the program, aspects of the program that made it work either better or worse and making the program more interesting and enjoyable for future participants	90% of group participants and 98% of eBody Project participants completed surveys. Clinician and peer-led group participants reported the group setting, feeling that they were not alone, and the letter exercise as most valuable. The most common response to what was most valuable for eBody Project participants was letters (19%), learning about the thin-ideal (12%), increased body image awareness (8%), the mirror exercise and role-plays (each 7%), the convenience (6%). To make eBody Project more interesting and enjoyable: having more support (20%), incorporating videos (13%), including more facts about EDs (6%) and having more engaging video- games and modules (5%)
Serdar et al. [69]	RCT	Females at high-risk for EDs n = 107 (face-to-face DB n = 112 (online DB) n = 114 controls	Face-to-face DB: 3, 1-h sessions, program based off manual includes interactive exercises that guide participants toward taking a stance against the thin-ideal Online DB: accessed via a website with content similar to the manual, three 1-h group discussions exclusively via text; moderated and synchronous; also had asynchronous components Control: completed measures but received no other materials	EDDS, IBSS-R, BES	Participants in both conditions manifested less body dissatisfaction at post-test compared with assessment-only participants; there were no significant differences in outcomes between the two modes of program delivery. No changes in thin-ideal internalization or ED symptoms among members of either active condition
Stice et al. [62]	RCT	Females with high body image and/or body dissatisfaction concerns n = 39 Body Project group n = 19 eBody Project n = 29 educational video control n = 20 educational brochure control	eBody Project: 6 online modules with activities designed to critique the thin-ideal over the course of 3 weeks; each module took between 30–40 min to complete Group Body Project: facilitator-led group sessions with exercises in which they critiqued the thin-ideal for four 1-h group sessions held on a weekly basis and in home exercises. Educational video condition: watched Dying to be Thin (55 min documentary) Educational brochure control condition: participants received a 2-page brochure by NEDA	IBSS-R, Satisfaction and Dissatisfaction with Body Parts Scale, DRES, BDI, EDDI	Internet participants showed reductions in ED risk factors and symptoms relative to the 2 control conditions at 1- and 2-year follow-up, but effects for in-person BP were greater than for eBP. The Internet intervention produced large weight gain prevention effects relative to the two control conditions at 1- and 2-year follow-up. Although the effects for the Internet versus group intervention were similar at post-test, results suggest that the effects faded more quickly for the Internet intervention
Stice et al. [61]	RCT	Females with high body image and/or body dissatisfaction concerns n = 39 Body Project group n = 19 eBody Project n = 29 educational video control n = 20 educational brochure control	eBody Project: 6 online modules with activities designed to critique the thin-ideal over the course of 3 weeks, 30–40 min per module Group Body Project: exercises critiquing the thin-ideal ideal during 4, 1-h group sessions held on a weekly basis and with homework Educational video: Dying to be Thin (55 min video) Educational brochure control condition: received a 2-page brochure by NEDA	IBSS-R, Satisfaction and Dissatisfaction with Body Parts Scale, DRES, BDI, EDDI	Internet and group BP participants showed greater pre-post reductions in ED risk factors and symptoms than video controls (M *d* = .47 and .54 respectively) and brochure controls (M *d* = .75 and .72, respectively), with many effects reaching significance. Effects did not differ significantly for Internet (eBody Project) versus group (Body Project group) participants. Acceptability of the Internet prototype was high, with 17/19 users (89%) completing all 6 modules. eBody project only produced a moderate reduction in thin-ideal internalization and ED symptoms (not significant) compared to brochure or video controls

*RCT* randomized controlled trial, *DRES* Dutch restrained eating scale, *PANAS-X* positive affect and negative affect scale-revised, *EDDI* eating disorder diagnostic interview, *SAS* social adjustment scale, *DBI-I* internet dissonance-based intervention, *CBI-I* internet cognitive-behavioural intervention, *BSQ* body shape questionnaire, *IBSS* ideal body stereotype scale, *EAT* eating attitudes test, *BDI-II* Beck depression inventory-2, *RED* reward-based eating drive, *ED* eating disorder, *DB* dissonance-based, *EDDS* eating disorder diagnostic scale, *IBSS-R* ideal body stereotype scale-revised, *BES* body esteem scale, *BDI* Beck depression inventory

One RCT studied an internet dissonance-based intervention (DBI-I; n = 90), internet cognitive-behavioural intervention (CBI-I; n = 88) and no intervention (n = 93) [67]. At post-intervention, both DBI-I and CBI-I led to greater reductions in body dissatisfaction, thin-ideal internalization, and depression than no intervention, but CBI-I was effective at reducing dieting and eating pathology relative to no intervention. In a secondary analysis of this RCT, the results suggest that DBI-I is a more effective strategy for reducing reward-based eating drive relative to the no intervention group, as the CBI-I intervention had no effect on reward-based eating drive [68]. Another RCT compared a similar online dissonance-based intervention (online DB; n = 112) to a face-to-face dissonance-based intervention (face-to-face DB; n = 107), and a control group (n = 114) [69]. Body dissatisfaction improved among participants in face-to-face and online DB compared to controls at post-test (no significant differences), however thin-ideal internalization and ED symptoms did not change in either active condition (Table 13).

#### Psychoeducation programs

*Children and adolescents* One open trial studied a general student sample (n = 453) using *ProYouth* [70]. The study demonstrated the impact that *ProYouth* has on early intervention and help-seeking among adolescents. Within three months of participation, 9.5% of participants took up treatment, 7.8% intended to start treatment, and 43.1% of the remaining sample reported that they would seek professional help if needed (Table 14).

**Table 14:** Psychoeducation programs (*with* discussion groups) for children and adolescents (< 18 years)

Reference	Type of study	Sample size	Intervention	Outcomes	Results
Moessner et al. [70]	Open trial	Openly accessible to all, but those reporting a risk for EDs or slight symptoms are encouraged to register n = 453	ProYouth: internet-based program for ED prevention and early intervention. Aims to inform about EDs and improve mental health literacy, to prevent ED development, and refer early to professional healthcare if needed. Has comprehensive psycho-educative materials, moderated forums, psychologist-led group chat sessions, blogs; can also book individual chat sessions with a counsellor	WCS, PHQ-4, Questions regarding use of ED services in the future	Within 3 months of participation, 43 (9.5%) took up treatment, 32 (7.8%) intended to start treatment, and 163 (43.1%) of the remaining reported that they would seek professional help in case of need (potential help-seeking). Approximately 50% of (potential) help-seekers stated that participation in ProYouth has changed their attitude towards help- seeking

*ED* eating disorder, *WCS* weight concerns scale, *PHQ-4* patient health questionnaire-4

*Emerging adults* One open trial studied the impact of *Appetite for Life* among a general sample of college students (n = 34), where 20.6% indicated that they gained knowledge about EDs by participating in the program, 14.7% said participation helped to clarify their questions, and 8.8% stated that they would not have known who to share disordered eating concerns with without the program [71] (Table 15).

**Table 15:** Psychoeducation programs (*with* discussion group) for emerging adults (18–25 years)

References	Type of study	Sample size	Intervention	Outcomes	Results
Lindenberg et al. [71]	Open trial	General sample of college students n = 34 actively used program n = 8 moderate risk n = 25 high risk n = 1 mild symptoms	Appetite for Life: Internet-based program for the prevention of EDs. It is a translation of German program Es[s]prit. Contains 5 modules of increasing intensity. First involves screening for level of impairment and ED risk. Based on screen results (low to high risk), users are assigned a specific module. Program contains psychoeducation and moderated forum. Option to connect with counsellor online for 30 min or referred to face-to-face counselling if more intensive services are needed	WCS, SEED, CR-EAT, EDE-Q Weekly monitoring questionnaire measures correlates of ED on four dimensions: 1) body dissatisfaction, 2) over concern with body weight and shape, 3) unbalanced nutrition and dieting, and 4) binge eating and compensatory behaviors, acceptability, feasibility	7 out of 34 (20.59%) indicated that they had gained knowledge on ED by participating, 5 (14.71%) said that their participation had helped them to clarify certain questions, 3 (8.82%) said that without it, they would not have known who to share their problems with, and 12 (35.29%) indicated that overall, they were pleased with it. 15 out of 34 (44.12%) said that it was ‘helpful’. The concepts of the various components were rated more positively by the “moderate risk” group, whereas participation in Appetite For Life in general was rated more positively by “high risk” participants
Minarik et al. [72]	Open trial	General population of young adult users n = 173 completed satisfaction survey	ProYouth: internet-based program for ED prevention and early intervention. Aims to inform about EDs and improve mental health literacy, to prevent ED development, and refer early to professional healthcare if needed. Has comprehensive psycho-educative materials, moderated forums, psychologist-led group chat sessions, blogs; can also book individual chat sessions with a counsellor	WCS, SEED, satisfaction survey	The most important reasons given for using ProYouth were anonymity and the possibility of free advice. 73% rated the statement "Overall, I am satisfied with ProYouth" as applicable. 70% would recommend ProYouth to friends if they were worried about their eating habits. 22% stated that without ProYouth they would not have known who to talk to about their questions and problems. Dissemination of the program must be actively pursued. An active, outreach form of dissemination (e.g., via workshops in schools) has proven its worth; approx. two thirds of participants stated that they had heard about ProYouth at school
Ali et al. [73]	RCT	Young adults at-risk for an ED (> 57 score on WCS) n = 17 ProYouth OZ (without peer-to-peer support) n = 17 ProYouth OZ Peers (with peer-to-peer support) n = 16 waitlist control	ProYouth OZ: adapted version of ProYouth, aiming to target young adults at high-risk for developing an ED; similarly comprised of psychoeducational information as well as a monitoring and feedback system (e.g., questions assessing body dissatisfaction, overconcern with weight/shape, etc.). Delivered over 6 weeks ProYouth OZ Peers: included all components of ProYouth OZ, but also included an online peer-to-peer support component, where participants were encouraged to attend weekly 1-h group chat sessions (with 4–6 participants) led by a peer with a lived ED experience (in the presence of a trained health professional). Chat sessions discussed body image, coping strategies, help-seeking pathways, etc	EDE-Q; help-seeking barriers, attitudes, intentions, behaviours, body image, quality of life, social support, loneliness, self-esteem, depression, anxiety, and stages of changes assessed via questionnaires; satisfaction with the intervention (e.g., asked “overall how satisfied were you with the chat session today?”)	Outcome data was limited (n = 15 completed a post-intervention assessment), and therefore it was not possible to conduct group comparisons or to test for any pre- to post-intervention changes between the groups over time. Individual outcome profiles of participants in each intervention (where pre- and post-intervention data was available) were examined. In ProYouth OZ, 1 (of 6) participant had decreased ED symptoms, and in ProYouth OZ Peers, both (2 of 2) participants reported decreased ED symptoms immediately after the intervention. Of the 6 participants in ProYouth OZ, 1 reported being very satisfied with the program. Of the 2 participants in ProYouth OZ Peers, both were very satisfied with the program

*ED* eating disorder, *WCS* weight concerns scale, *SEED* short evaluation of eating disorders, *CR-EAT* clinical and research inventory for eating disorders, *EDE-Q* eating disorder examination questionnaire

One open trial studied *ProYouth* in a general population of young adults (n = 173), where 22% stated that without the program, they would not have known who to share their concerns or questions with regarding disordered eating [72]. One RCT studied an adapted version of *ProYouth*, aiming to specifically target young adults at high-risk for developing an ED (based on Weight Concerns Scale [WCS] score); participants were randomized to either *ProYouth OZ* (n = 17), *ProYouth OZ Peers* (included peer support; n = 17) or waitlist control (n = 16) [73]. More participants in *ProYouth OZ Peers* showed a decrease in disordered eating than those in *ProYouth OZ* immediately after the intervention, although there was limited outcome data, so strong conclusions were not possible (Table 15).

### Other e-technology prevention

#### Mobile applications (‘apps’)

*Emerging adults* One RCT randomized a group of students to immediate use of *GGBI Body Positive* app (n = 25), or use of the app 16 days later (n = 25) [74]. Compared to those that used the app later, immediate use showed a greater decrease in body dysmorphic disorder symptoms and body dissatisfaction. Though not significant, the desire to be thin and risk for developing an ED decreased in both groups after using the app (Table 16).

**Table 16:** Other e-technology (including mobile apps and text messaging interventions) for emerging adults (18–25)

References	Type of study	Sample size	Intervention	Outcomes	Results
Cerea et al. [74]	RCT	Female university students at high-risk of body image disorders n = 25 immediate use of app (iApp) n = 25 delayed use of app (dApp)	GGBI Positive Body Image App: cognitive-behavioural exercises to challenge maladaptive beliefs associated with body dissatisfaction and body image disorders. Participants had to complete 3 min of the app each day immediately for 16 consecutive days Delayed use: started using app 16 days after immediate group, for 16 consecutive days (3 min/day)	EDI-3, DASS-21, QDC (to assess body dissatisfaction)	Significant group (iApp vs. dApp) x Time (baseline vs. first 16 days) interaction on body dissatisfaction and body dysmorphic disorder symptoms (medium effect sizes), where iApp group showed greater decrease from baseline to first 16 days compared to dApp group. Pertaining to ED symptoms (EDI-3), no significant Group x Time interaction was detected, however, some reductions (not significant) emerged with respect to desire to be thin and for the risk of developing EDs in iApp and dApp groups following GGBI use
Rodgers et al. [75]	RCT	Young adults from high schools, youth organizations, or university n = 237	BodiMojo: mobile phone app with (1) intervention messages sent twice daily; (2) mood tracking and emotion regulation; (3) gratitude journaling. Intervention messages were based on self-compassion, media literacy, healthy lifestyle-related content. The daily intervention messages came in the form of an affirmation, a behavioral tip, or psychoeducation, and some contained a link to a quiz or an audio meditation	PACS, SCS, BES (appearance esteem subscale), BI-AAQ	In comparison to the control group, participants who used the BodiMojo intervention reported improved appearance esteem and self-compassion. In contrast, significant effects were not found for body image flexibility. No improvements in appearance comparison or mood
Fioravanti et al. [76]	RCT	Young adult women n = 41 Body Positive profiles n = 41 Fitspiration profiles n = 40 neutral profiles	Participants were asked to follow their assigned profiles (on Instagram, created by researchers) for 28 days; they saw 1 new post/day and 3 Instagram stories/day Body Positive profile: had to follow and view images related to hashtags including: #BodyPositive, #BoPo, #ShowUs, #normalizeNormalBodies, #BodyPositive, #loveyourbody Fitspiration profile: had to follow and view images related to hashtags including: #fitspiration, #fitmodel, #fitmotivation, #body transformation #fitspogirl; fitspiration = sustaining or improving health and fitness Neutral profiles: had to follow and view images related to hashtags including: #animals, #nature, #travel, #landscape	C-VAS (state mood and body satisfaction), SACS, PACS, MBSRQ-AS	Daily exposure to body positive images was associated with the highest rate of growth of positive mood and body satisfaction (vs. those exposed to fitspiration or neutral content). Daily exposure to fitspiration images was associated with the highest rates of growth of negative mood and appearance comparison (vs. those exposed to body positive images). There was no difference in the growth of negative mood and body satisfaction between participants exposed daily to fitspiration and neutral content
Smith et al. [77]	Open trial	Female undergraduate students with high levels of body checking behaviours n = 44	5 text messaging interventions for body checking (1/day for 5 days): (1) Psychoeducation- “think of a time in your life when you were checking your body a lot… Reflect on how you felt”; (2) Visualization- “Imagine yourself standing in front of a mirror wearing only a swimsuit… how is your mood/feelings?”; (3) Behavioural- “identify 2 checking urges that you will attempt to resist by using deep breathing”; (4) Cognitive- “What could you say to yourself next time you have an unwanted urge to check?”; (5) Cognitive dissonance- “What would you tell your friend about her checking behaviours?”	BCCS, BSQ, BIAQ, BCQ, SATAQ-3, Body Checking Behaviours measure (number of times per day engaged in weighing themselves, feeling thighs for fatness, sucking in stomach, feeling/pinching stomach to measure fatness, comparing body to others, checking body size in mirror, checking fat for jiggling, checking to see if thighs spread while sitting)	In total, 1804 text messages were sent to the 44 participants. Pretest to post-test analyses found healthy improvements (significantly decreased scores) in BSQ, BCQ, BIAQ, BCCS, SATAQ-3 (large to medium effect sizes) number of attitudes and behaviours related to body checking were positively impacted. Body checking behaviours increased within each day (highest checking behaviours at night) but decreased across the 5-day intervention period. Overall, brief intervention led to reduced body checking behaviours and led to higher body satisfaction

*RCT* randomized controlled trial, *iApp* immediate use of app, *dApp* delayed used of app, *GGBI* GGApps body image app, *EDI-3* eating disorder inventory-3, *DASS-21* depression, anxiety, stress scale-21 items, *QDC* questionario sul dismorfismo corporeo (‘body dysmorphic questionnaire’), *ED* eating disorder, *PACS* physical appearance comparison scale, *SCS* self-compassion scale, *BES* body esteem scale, *BI-AAQ* body image-acceptance and action questionnaire, *C-VAS* computer-based visual analogue scale, *SACS* state appearance comparison scale, *MBSRQ-AS* multidimensional body-self relations questionnaire-appearance scales, *BCCS* body checking cognitions scale, *BSQ* body shape questionnaire, *BIAQ* body image avoidance questionnaire, *BCQ* body checking questionnaire, *SATAQ-3* sociocultural attitudes towards appearance scale-3

One RCT recruited young adults (n = 237) to an intervention group where participants had to use the *BodiMojo* app (based on the website version [55]), or a control group [75]. Participants in the *BodiMojo* group reported improved appearance esteem and self-compassion compared to controls. (Table 16).

One RCT involving young adult women studied daily exposure to Instagram app profiles (created by the research team) with either body positive (n = 41), fitspiration (n = 41), or neutral (n = 40) images and related hashtags [76]. Overall, exposure to body positive content had the highest rates of growth for positive mood and body satisfaction, whereas exposure to fitspiration content had the highest rates of growth of negative mood and appearance comparison (Table 16).

#### Text messaging prevention

*Emerging adults* One open trial invited students with high levels of body checking (n = 44) to take part in a text messaging intervention, involving one text message sent per day for five days [77]. A positive impact of the intervention was observed-across the five-day intervention period, there was an overall decrease in body checking behaviours and body satisfaction increased (Table 16).

### Computer-based prevention

#### Cognitive-behavioural programs

*Children and adolescents* One RCT randomized adolescent girls at high-risk for EDs (based on WCS score) to either *AcceptME*, intervention (n = 62) or waitlist control (n = 30) [78]. Compared to controls, participants in the intervention group had significantly lower weight and shape concerns at the end of the program, with effects maintained at 1-month follow-up. ED risk also decreased amongst *AcceptME* participants compared to controls (Table 17).

**Table 17:** Cognitive-behavioural programs (*without* discussion groups) for children and adolescents (< 18 years)

Reference	Type of study	Sample size	Intervention	Outcomes	Results
Karekla et al. [78]	RCT	High school and university female students showing signs and symptoms of an ED and at high-risk for an ED (> 52 score on WCS) n = 62 AcceptME intervention n = 30 waitlist control	AcceptME: digital gamified Acceptance and Commitment Therapy (ACT; based in CBT that targets ineffective internal control strategies and inflexibility) early-intervention program consisting of 6 sessions (30 min each). AcceptME portrayed the story of a young girl contemplating whether to enter a reality television fashion contest. Participants followed the main character through making the decision and faced situations eliciting common challenging situations, thoughts, and emotions related to body image. Participants completed exercises in the program aiming to teach and apply ACT skills (e.g., acceptance, present moment awareness, values-consistent living)	WCS, EDDS, EDE-Q, YQOL-SF, BSQ-8C, BI-AAQ, BIAQ	Users of AcceptME had significantly lower EDE-Q weight and shape concerns at end-of-intervention vs. waitlist controls, with large effects; effects appeared to be maintained at 1-month follow-up. Most participants scored below the at-risk cut-off (WCS score < 52) in the AcceptME group at end-of-intervention (57.1%) vs. controls (7.1%), with odds of falling into the at-risk group being 14.5 times higher for controls. At 1-month follow-up, 72% of completers reported scores below the at-risk cut-off in AcceptME. The main effect of time was statistically significant for BSQ-8C (body dissatisfaction) and BI-AAQ and BIAQ (body image) between baseline, end-of-intervention, and/or 1-month follow-up scores. There was no significant time effect for quality of life

*RCT* randomized controlled trial, *ED* eating disorder, *WCS* weight concerns scale, *ACT* acceptance and commitment therapy, *CBT* cognitive behavioural therapy, *EDDS* eating disorder diagnostic scale, *EDE-Q* eating disorder examination questionnaire, *YQOL-SF* youth quality of life instrument-short form, *BSQ-8C* body shape questionnaire (8 question), *BI-AAQ* body image-acceptance and action questionnaire, *BIAQ* body image avoidance questionnaire

*Emerging adults* One RCT studied participants in the *Food, Mood, and Attitude* (FMA) program (n = 116) versus a control group (n = 115) [79]. FMA participants improved on all ED symptom-related measures compared to controls from baseline to 3-month follow-up. A different RCT compared FMA and MyStudentBody.com-Nutrition (total n = 32) to a non-eating related website control (n = 30) [80]. Unlike the previous RCT, the intervention did not produce any significant effects related to ED symptoms (Table 18).

**Table 18:** Cognitive-behavioural programs (*without* discussion groups) for emerging adults (18–25 years)

References	Type of study	Sample size	Intervention	Outcomes	Results
Franko et al. [79]	RCT	Female university students at-risk or low risk for EDs n = 116 Food, Mood, and Attitude n = 115 Control	FMA: 2-h CD-ROM addressing ED risk factors (perceived pressure to be thin, thin-ideal internalization, body dissatisfaction, dieting, negative affect); based on cognitive-behavioural and interpersonal/relational theories	Program satisfaction, EDE-Q, SATAQ, Knowledge Test, Q-EDD	FMA group improved on all measures relative to controls. Significant time x condition x risk status interactions were found on measures of internalization of sociocultural attitudes about thinness, shape and weight concerns, indicating that at-risk participants in FMA improved to a greater extent than did low-risk participants from baseline to 3-month follow-up. At 3-month follow-up, fewer women in intervention reported overeating and excessive exercise relative to controls. 97% of students were ‘very satisfied’ or ‘extremely satisfied’ with program
Franko et al. [80]	RCT	Female, Latina university students n = 32 to either Food, Mood, and Attitude or MyStudentBody n = 30 Control	FMA: 2-h CD-ROM addressing ED risk factors (perceived pressure to be thin, thin-ideal internalization, body dissatisfaction, dieting, negative affect); based on cognitive-behavioural and interpersonal/relational theories MyStudentBody.com-Nutrition: website to provide nutrition education Control: 2 non-eating-related websites	Stages of dietary and physical activity change questionnaire, BSQ, SATAQ	BSQ scores decreased from pre to post-test only for intervention groups at the level of a trend (not significant). SATAQ data indicated no intervention effects (both interventions and control groups decreased scores over time). Some promise in decreasing body dissatisfaction and increasing fruit and vegetable intake among Latina students via 2 computer-based interventions
Fitzsimmons-Craft et al. [81]	RCT	Females screening as high-risk for an ED n = 352 Body Positive chatbot intervention n = 348 delayed control	Body Positive chatbot: fully automated, conversation-based, cognitive-behavioural computer program, simulating human conversation, and designed to improve body image. It was based on Student Bodies (SB), similarly addressing topics like the thin- ideal and media literacy, but interactions were synchronous. Conversations based on these topics were programmed into the chatbot and the chatbot initiated conversations with users. Participants were to complete 2 conversations (sessions) per week (10 min each) for 1 month Delayed control: provided access to the chatbot 6 months later	WCS, SATAQ (thin/low body fat subscale), EDE-Q, PHQ-8, GAD-7, helpfulness (e.g., asked “did you find that conversation helpful today?”)	There was a significantly greater reduction in weight/shape concerns in the chatbot intervention vs. control at 3-month (*d* = -0.20; *p* = 0.03) and 6-month (*d* = -0.19; *p* = 0.04) follow-up. There were no differences in change in thin-ideal internalization, depression, or anxiety. Compared to controls, the intervention was associated with significantly greater reductions in overall ED psychopathology at 3-months (*d* = -0.29; *p* = 0.003), but not 6-month follow-up. The odds of remaining nonclinical for EDs were significantly higher in intervention vs. control at both 3- (OR = 2.37, 95% CI [1.37, 4.11]) and 6-month follow-ups (OR = 2.13, 95% CI [1.26, 3.59])

*RCT* randomized controlled trial, *FMA* food, mood, and attitude, *CD-ROM* compact disk-read only memory, *ED* eating disorder, *EDE-Q* eating disorder examination questionnaire, *SATAQ* sociocultural attitudes towards appearance scale, *Q-EDD* questionnaire for eating disorder diagnoses, *ED* eating disorder, *BSQ* body shape questionnaire, *SB* student bodies, *WCS* weight concerns scale, *PHQ-8* patient health questionnaire-8, *GAD-7* generalized anxiety disorder-7, *OR* odds ratio, *CI* confidence interval

One RCT, involving females at high-risk for an ED (based on WCS score), randomized participants to a chatbot intervention (n = 352) or a delayed control group (n = 348) [81]. Intervention participants had significantly greater reductions in weight and shape concerns versus controls at 3- and 6-month follow-up. The results also suggest that the chatbot intervention may reduce ED onset (Table 18).

#### Psychoeducation programs

*Children and adolescents* One RCT involved middle school students randomized to *Trouble on the Tightrope: In Search of Skateboard Sam* (n = 92), or a control group (n = 98) [82]. Participants in the intervention group for whom puberty was underway showed greater improvements in body esteem from baseline to post-test, relative to controls (Table 19).

**Table 19:** Psychoeducation programs (*without* discussion groups) for children and adolescents (< 18 years)

References	Type of study	Sample size	Intervention	Outcomes	Results
Cousineau et al. [82]	RCT	Middle school students (11–12 years old) n = 92 intervention n = 98 placebo control	Trouble on the Tightrope: In Search of Skateboard Sam (www.skateboard-sam.com): fully animated computer program on puberty, nutrition, physical activity, self-esteem, and peer relations. Each 15 min module offers targeted information based on a short 14-item profile quiz taken by each participant assessing relevant characteristics (e.g., gender)	Puberty knowledge questionnaire, SPP, BES	Participants in intervention group increased their scores on the puberty knowledge questionnaire slightly from baseline to post-test and maintained this increase at 3-month follow-up vs. controls (not significant). Participants in the intervention group for whom puberty was underway showed improvement on the Weight subscale of BES from baseline to post-test, relative to control group, small effect. On the Global Self-Worth subscale, girls in the intervention condition improved their scores from baseline to post-test vs. controls. Girls in the intervention group showed a similar improvement on the total SPP score from baseline to post-test as vs. control girls
Withers et al. [83]	RCT	Grade 7 girls n = 104 videotape Intervention n = 114 Control	22-min videotape: (1) determinants of body size, variation in ‘‘normal’’ female appearance, puberty; (2) historical and sociocultural influences on female appearance, media’s role in shaping this ideal; (3) the negative effects of extreme dieting, EDs and consequences, emotional eating; (4) healthy eating habits, the importance of healthy eating; and (5) creating a healthy body image and boosting self-image	Need for Cognition Scale (shortened), PII (shortened), Contour Drawing Rating Scale, EDI (body dissatisfaction and drive for thinness subscales), pre and post knowledge questionnaire; to measure dieting behaviour, participants were asked questions such as “how often have you dieted?”, “how likely are you to go on a weight loss diet in the future?”	Findings demonstrated that compared to a non-intervention control group, girls who watched the prevention videotape made small, but significant, positive changes in drive for thinness and intention to diet and had improved scores on knowledge items, not maintained at follow up

*RCT* randomized controlled trial, *SPP* self-perception profile for adolescents, *BES* body esteem scale, *PII* personal involvement inventory

One RCT recruited grade 7 girls to a puberty videotape intervention (n = 104) or control group (n = 114) [83]. Compared to controls, girls that watched the video had significant improvements in drive for thinness, intention to diet, and knowledge regarding body image and puberty, but this was not maintained at 1-month follow-up (Table 19).

### Online caregiver prevention interventions focused on child outcomes

#### Caregiver-focused prevention for caregivers of children and adolescents with disordered eating

*Children and adolescents* One pilot open trial recruited girls at-risk (n = 12) or at high-risk (n = 22) of developing Anorexia Nervosa along with their parents/guardians to take part in *Parents Act Now;* Anorexia Nervosa risk level was based on pre-determined screening criteria, where those at-risk exhibited high levels of weight/shape concerns and perfectionism, and those at high-risk exhibited specific weight-related criteria in combination with high levels of weight/shape concerns and/or perfectionism [84]. Parents of high-risk children actively used the program to a greater extent than parents of at-risk children, however both groups demonstrated improvements in early symptoms and risk factors, ED attitudes and behaviours, and remained stable or increased in body weight from pre- to post-intervention. One RCT studied a similar population and the German version of *Parents Act Now* (Eltern als Therapeuten) [85]. Between pre-intervention and 12-month follow-up, girls in the intervention (n = 32) gained significantly more weight than waitlist controls (n = 34) (Table 20).

**Table 20:** Caregiver-focused prevention for caregivers of children and adolescents (< 18 years)

References	Type of study	Sample size	Intervention	Outcomes	Results
Jones et al. [84]	Open trial (pilot)	Children at-risk of AN n = 46 (12 at risk (R), 22 at high-risk (HR), 12 ED diagnosed)	Parents Act Now (P@T or Elternals Therapeuten E@T-the German version): based on first phase of family-based treatment. 6 online sessions (6 weeks), moderated by ED experts. Includes discussions, journals related to weight, eating, and exercise, videos, and 2 phone calls for individualized feedback. Sessions focused on activating parental concern, empowering parents to take action, providing education and practical approaches for intervening in problematic behaviours, and providing guidance about ongoing monitoring and maintenance of progress	Feasibility, acceptability, PvA scale, WCS, EDE-Q, EDE, EDI-2, FMPS, RSES	HR parent participants logged on more frequently and viewed more sessions than R parents 19 parents completed post-assessment and rated the overall program quite favourably, and reported that they would highly recommend the program. Overall improvements in those in R and HR categories: improvements in risk factors and early symptoms between initial screening and post-assessment; participants remained stable or increased in ideal body weight and reported decreased ED attitudes and behaviours
Jacobi et al. [85]	RCT	Children at-risk of Anorexia Nervosa n = 32 (E@T) n = 34 (control)	Elternals Therapeuten E@T-the German version: based on first phase of family-based treatment. 6 online sessions (6 weeks), moderated by ED experts. Includes discussions, journals related to weight, eating, and exercise, videos, and 2 phone calls for individualized feedback. Sessions focused on activating parental concern, empowering parents to take action, providing education and practical approaches for intervening in problematic behaviours, and providing guidance about ongoing monitoring and maintenance of progress	Expected body weight % change, WCS, EDE, EDI-2	Low intervention acceptance/use, but participating parents rated the program quite favorably as “good” and would recommend to other parents. Of the primary outcomes, only 1 significant difference between the intervention and the control group was found: between preintervention and 12-month FU, girls of the intervention group gained significantly more weight more quickly as indicated by change in percentage of EBW compared with girls in the control group. Parent-reported reasons for unwillingness to participate: The majority of these parents responded that they did not perceive the identified risk factors and early symptoms in their daughters as problematic and participation in a preventive intervention not necessary
Bruning Brown et al. [86]	Controlled trial	Females in high school physical education/ health class Students: n = 102 SB n = 51 comparison Parents: n = 22 intervention n = 47 waitlist control	Student Bodies student intervention: structured 8-week internet- and cognitive-behavioural based intervention including an asynchronous, moderated discussion group and psychoeducation Student Bodies parent intervention: unstructured web-based program that parents had 4 weeks to complete; includes an online discussion group. Program encourages parents to accept variations in weight and shape and to discourage negative attitudes and behaviors	EDI (drive for thinness and bulimia subscales), EDE-Q (weight, shape, and restraint subscales), WCS, Knowledge Test, PACS	Students using the program reported significantly reduced eating restraint and had significantly greater increases in knowledge than did students in the comparison group from baseline to post-assessment. No significant differences at 3-month follow-up. Parents in the intervention significantly decreased their overall critical attitudes toward weight and shape compared to parents in control group from baseline to post intervention (note: parents did not do 3-month follow-up like students did)
Diedrichs et al. [87]	RCT	Mothers who identified body image issues among daughters n = 53 dyads (website-unstructured) n = 57 dyads (website-tailored) n = 48 dyads (assessment-only control)	Dove Self Esteem Project Website: online information hub designed to provide mothers with information and tools to help foster positive body image among their daughters and themselves. Website-unstructured condition: mothers browsed the website without guidance. Website-tailored condition: mothers were provided with a written personalized pathway that guided them. Control: assessment only, did not view website nor receive a website link	BES (appearance esteem and weight esteem subscales), SATAQ-3, Perceived Sociocultural Pressures Scale, Social Comparison to Models and Peers scale, RSES, frequency of mother-daughter conversations about body image, negative affect, maternal pressures, help seeking behaviours, adherence	Daughters in both website conditions had significantly engaged in more self-reported conversations with their daughters about body image at post-exposure and 6-week follow-up. At 12-month follow-up, mothers in the website-tailored condition were almost 3 times as likely than controls and just over 2.5 times more likely than mothers in the website-unstructured condition to report that they had sought additional support around body image for their daughters. Daughters whose mothers viewed the website had higher self-esteem and reduced negative affect at 6-week follow-up (not maintained at 12 months). There were no differences on daughters’ body image, and risk factors among mothers or daughters, at post-exposure or follow-up. Tailoring website content appeared beneficial

*AN* anorexia nervosa, *R* at risk, *HR* at high risk, *ED* eating disorder, *P@T* parents act now, *PvA* parents versus anorexia scale, *WCS* weight concerns scale, *EDE-Q* eating disorder examination questionnaire, *EDE* eating disorder examination, *EDI-2* eating disorder inventory-2, *FMPS* Frost multidimensional scale, *RSES* Rosenberg self-esteem scale, *PACS* physical appearance comparison scale, *BES* body esteem scale, *SATAQ-3* sociocultural attitudes towards appearance scale-3

One controlled trial studied high school females in *Student Bodies* (SB; n = 102) versus a comparison group (n = 51), as well as their parents participating in a parent version of SB (n = 22) versus a control group (n = 47) [86]. Students in SB reported significantly reduced eating restraint than students in the comparison group from baseline to post-intervention. Parents in the SB parent intervention significantly decreased their critical attitudes toward weight and shape compared to parents in the control group, from baseline to post-intervention (Table 20).

One RCT studied mother-daughter dyads completing either a website-unstructured (n = 53 dyads) or website-tailored intervention (n = 57 dyads), or an assessment-only control (n = 48 dyads) [87]. Mothers in both website conditions reported having significantly more conversations with their daughters about body image at post-test and 6-week follow-up, relative to controls. There were no differences in daughters’ body image and risk factors among mothers or daughters at post-test or follow-up (Table 20).

## Discussion

To our knowledge, this is the first scoping review identifying all evidence on virtual ED prevention focused specifically on children and adolescents (< 18 years) and emerging adults (18–25 years) at-risk for EDs. Our review identified 67 studies, examining asynchronous (n = 35) and synchronous (n = 18) internet-based programs, other e-technology including mobile apps (n = 3) and text messaging interventions (n = 1), computer-based programs (n = 6), and online caregiver interventions focused on child outcomes (n = 4). Few included studies focused on children and adolescents (n = 18), while the vast majority focused on emerging adults (n = 49). For children and adolescents, the most widely researched programs were *Student Bodies* and its adapted versions (n = 4), *virtual/eBody Project* (n = 2), and *Parents Act Now* (n = 2). For emerging adults, the most widely researched programs were *Student Bodies* and its adapted versions (n = 16), *eBody Project* (n = 6), and *Expand Your Horizon* (n = 4). The scoping review determined that a variety of evidence exists suggesting virtually delivered ED prevention programs can be effective at reducing ED symptoms and risk, and in some cases, demonstrate comparable efficacy to in-person delivery.

Virtual prevention interventions have been successfully implemented in other areas of mental health, specifically within suicide prevention. A systematic review on web- and mobile-based suicide prevention interventions for youth found that the interventions significantly reduced risk factors for suicide, including suicidal ideation, depression, and hopelessness in participants [88]. Reductions in risk for EDs were similarly found in our review, among children, adolescents, and emerging adults in various virtual prevention programs for EDs, including *Student Bodies* and *eBody Project*, which significantly reduced ED onset and symptomatology, as well as cognitions related to body image, weight, and shape. These effects were found across a range of measures, including the Eating Disorder Examination Questionnaire (EDE-Q) and Body Shape Questionnaire (BSQ). Online and social media-based interventions for suicide prevention have also demonstrated high feasibility and acceptability, with users reporting satisfaction with online group interventions at enhancing feelings of connectedness between young people [89]. Comparable findings were illustrated in our scoping review, as children, adolescents, and emerging adults in internet-based group chatrooms aiming to improve body image and eating behaviours reported a strong preference for online instead of face-to-face communication surrounding ED-related concerns [56–58].

With unprecedented increases in new ED diagnoses and behaviours during the COVID-19 pandemic among children, adolescents, and emerging adults worldwide [90–92], there is a clear need to rapidly implement effective ED prevention interventions to reduce the onset of illness. As services may never return to in-person delivery only, it is important to consider the use of evidence-based virtual ED prevention interventions, such as *Student Bodies* and *eBody Project* among children, adolescents, and emerging adults at-risk for EDs, as well as ensure that programs such as these are widely available and free of charge. Our scoping review determined that these programs and their adapted versions have the greatest evidence to suggest that they are successful at reducing ED symptoms, such as weight/shape concerns, body dissatisfaction, and internalization of the thin-ideal. When compared to in-person delivery, *Student Bodies* programs consistently improved ED signs and symptoms, while *eBody Project* was not always superior to its in-person delivered counterparts. However, it is evident that the use of virtually delivered ED prevention programs such as these provide some benefit to users, compared to those who do not use any program.

While our findings are particularly relevant given the current COVID-19 restrictions, they may also be important to consider for the post-pandemic era. Virtual delivery of mental health services, including telepsychiatry and telepsychology, has increased dramatically since March 2020, and is predicted to continue at a high rate in the post-pandemic era, once all restrictions are lifted [93, 94]. It is possible that continued use of virtual modalities for ED treatment may indicate a transition for virtual delivery for prevention services as well. Remote areas where in-person specialized care or services may be unavailable or inaccessible, may especially benefit from continued virtual delivery of ED treatment, as well as virtual prevention interventions.

### Strengths

The strengths of our review are numerous. We used a rigorous and evidence-based methodology for our scoping review, which included a thorough review of literature from seven databases, without language restrictions, and we had few exclusion criteria. We translated several papers into English for full-text review and examined the references of included reviews and book chapters to ensure we did not miss any relevant studies. We also conducted a forward citation chaining process to update our search. Additionally, we were able to include an abundance of high-quality data (47 RCTs and 3 meta-analyses) in the field of virtual prevention for our populations of interest. This data had large sample sizes as well as a wide variety of sample characteristics, including general populations, those with self-reported ED symptoms, and individuals deemed at-risk according to screening questionnaires.

### Limitations

Although thorough, our search strategy had limitations. We were unable to retrieve four citations as full-text articles, as they could not be located. Our evidence involving emerging adult populations was also more than double that of our evidence for child and adolescent groups, making it difficult to form strong conclusions for this younger age group. Many studies enrolled participants based on symptom evaluation using instruments rather than diagnostic interview, and as a result, some high-risk participants might have actually met diagnostic criteria for EDs. As rating the quality of evidence is not a component of scoping review methodology [11], our findings cannot comment on this. However, our work lays the foundation for a systematic review, which would involve an evaluation of the strength of the evidence and is a feasible and logical next step in advancing knowledge in this particular field. Despite these limitations, this review represents a significant step forward in understanding the types of virtual ED prevention programs that exist, as well as those with the greatest evidence base demonstrating the effects of these programs.

### Future directions

Several gaps were noted, which should be a focus for future study. First, more research on virtual prevention programs specifically for children and adolescents (< 18 years) is required. Second, large-scale studies of efficacy with long-term follow-up for ED prevention interventions, including text messaging, mobile apps, online cognitive restructuring, and online imagery rescripting interventions are needed. Evidence-based recommendations for virtual ED prevention interventions that have been reviewed and rated by a diverse consensus panel, similar to our virtual care recommendations for children, adolescents, and emerging adults during the COVID-19 pandemic and beyond [10] would also be beneficial so clinicians can direct individuals experiencing early signs and symptoms of EDs to effective prevention programs. As not all of the researched ED prevention programs are widely available and freely accessible, additional research on how to improve access to virtual ED prevention services for vulnerable populations (e.g., equity-seeking and marginalized youth) and gender diverse groups is also necessary.

## Conclusions

This scoping review identified a variety of evidence for ED prevention programs for children, adolescents, and emerging adults. For children and adolescents, the most widely researched programs were *Student Bodies* and its adapted versions, *eBody Project*, and *Parents Act Now*. For emerging adults, the most widely researched programs were *Student Bodies* and its adapted versions, *eBody Project,* and *Expand Your Horizon.* Most studies included emerging adults rather than children and adolescents, and therefore, additional research focusing on ED prevention interventions for those under 18 years of age is required to form stronger conclusions for this population. Future research should also be conducted to determine the long-term efficacy of several understudied ED prevention interventions, including text messaging, mobile apps, online cognitive restructuring, and online imagery rescripting programs, as well as evidence-based recommendations for virtual ED prevention for children, adolescents, and emerging adults at-risk for EDs. Research for improving access to virtual ED prevention services for vulnerable and gender diverse groups should also be prioritized.

## Supplementary Information

**Additional file 1**. Database search strategy.

## Data Availability

All data reported is published and in the public domain.

## Abbreviations

BIR: Body image rescripting
BMI: Body mass index
BP: Body project
BSQ: Body shape questionnaire
CBI-I: Internet cognitive-behavioural intervention
CBT: Cognitive-behavioural therapy
CD-ROM: Compact disk-read only memory
DB: Dissonance-based
DBI-I: Internet dissonance-based intervention
eBP: E-Body project
ED: Eating disorder
EDE-Q: Eating disorder examination questionnaire
EW: Expressive writing
FMA: Food, mood, and attitude
GIR: General image rescripting
HRHM: Higher-risk, higher-motivated
ICBT-P: Internet cognitive behaviour therapy program for perfectionism
ICBT-S: Internet cognitive behaviour therapy for nonspecific stress management program
MS-T: Media smart-targeted
PRISMA: Preferred reporting items for systematic review and meta-analysis
RCT: Randomized controlled trial
SB-AN: Student bodies-anorexia nervosa
SB-ED: Student bodies-eating disorders
SB2: Student bodies 2
SB2-BED: Student bodies 2-binge eating disorder
SB +: Student bodies +
vBP: Virtual body project
WCS: Weight concerns scale
zBMI: Standardized body mass index
