# Supplementary material for: Virtual prevention of eating disorders in children, adolescents, and emerging adults: a scoping review
Source: J Eat Disord. 2022 Jul 6;10:94. doi: 10.1186/s40337-022-00616-8 (PMC9258006; doi:10.1186/s40337-022-00616-8)
Supplement: Supplementary file 1 — Additional file 1. Database search strategy. [file 40337_2022_616_MOESM1_ESM.docx]

**Supplemental File 1.**

Database Search Strategy

--------------------------------------------------------------------------------

1 exp "Feeding and Eating Disorders"/ or eating disorder*.mp.

2 feeding disorder*.mp.

3 exp Anorexia Nervosa/ or Anorexia/ or anorexia.mp.

4 exp Bulimia Nervosa/ or Bulimia/ or bulimia.mp.

5 food avoidance.mp.

6 food intake disorder*.mp.

7 ((avoidant or restrictive) adj2 food adj3 disorder*).mp.

8 hyperphagia.mp. or Hyperphagia/

9 food aversion.mp.

10 binge eat*.mp.

11 (bulimi* or anorexic?).mp.

12 purging.mp.

13 food refusal.mp.

14 feeding related disorder*.mp.

15 eating related disorder*.mp.

16 (binge adj2 eat*).mp.

17 (purg* adj2 eat*).mp.

18 ((avoid* or restrict*) adj2 food adj3 disorder*).mp.

19 disordered eating.mp.

20 eating disorder*.mp.

21 or/1-20

22 exp Body Image/ or body image*.mp.

23 (body adj2 dissatisfaction*).mp.

24 (body adj2 (identity or schema* or perception*)).mp.

25 (eating concern* or shape concern* or weight concern*).mp.

26 dietary restraint*.mp.

27 21 or 22 or 23 or 24 or 25 or 26

28 computer assisted therapy.mp. or exp Therapy, Computer-Assisted/

29 (proyouth or pro youth or ebody or vbody or vbp or student bodies or media smart).mp. (459)

30 everybody.mp.

31 (food mood adj2 attitude*).mp.

32 healthy body image program*.mp.

33 or/28-32

34 27 and 33

35 exp Telemedicine/

36 Telenursing/

37 Remote Consultation/

38 internet/ or internet access/ or "internet of things"/ or internet-based intervention/

39 exp Programmed Instruction as Topic/

40 exp Computers, Handheld/

41 Mobile Applications/

42 exp Cell Phone/

43 ((sms or mms or text) and messag*).tw.

44 exp telecommunications/ or electronic mail/

45 email*.mp.

46 (((mobile* or cell or smart*) and phone*) or smartphone* or (smart adj3 (technolog* or device*))).mp.

47 (ios or android).ti,ab,kf.

48 (ipad* or iphone* or ipod*).ti,ab,kf.

49 (tablet* or computer*).ti,ab,kf.

50 ((online or web* or virtual) and (education* or train*)).ti,ab,kf.

51 personal digital assistant*.ti,ab,kf.

52 Remote Sensing Technology/

53 exp Telephone/

54 exp Technology/

55 (telemetry or telehealth* or tele health* or telehomecare* or tele homecare* or telecoach* or tele coach* or telecommunication* or videoconference* or video conferenc* or videoconsult* or video consult* or teleconference* or tele conference* or telecare* or tele care* or ehealth or e health).mp.

56 (teleassist* or tele assist* or telebased or tele based or telederm* or tele derm* or telediagno* or tele diagno* or teleecho* or tele echo* or teleemerg* or tele emerg* or telefollow* or tele follow* or teleguid* or tele guid* or telehealth* or tele health* or telehome* or tele home* or teleinterven* or tele interven* or telemanag* or tele manag* or telemed* or tele med* or telemental* or tele mental*).mp.

57 (telemonitor* or tele monitor* or telenurs* or tele nurs* or telepalliat* or tele palliat* or teleprocedu* or tele procedu* or telepsych* or tele psych* or telerefer* or tele refer* or telerehab* or tele rehab* or telesurger* or tele surger* or telesurgic* or tele surgic* or teletreat* or tele treat* or teletriage or tele triage or telehelp* or tele help*).mp.

58 (telepract* or tele pract* or teletherap* or tele therap* or teleconsult* or tele consult* or telecounsel* or tele counsel* or telespecialist* or tele specialist* or telesupport* or tele support* or tele supervis* or telesupervis* or televisit* or tele visit* or telesen* or tele sens*).mp.

59 (telecancer or tele cancer or tele cardiolo* or telecardiolog* or teledental or tele dental or telederm* or tele derm* or teledialysis or tele dialysis or teleepileps* or tele epileps* or teleICU or tele ICU or teleoncolo* or tele oncolo* or teleopthalm* or tele opthalm* or telepalliat* or tele palliat* or tele patholog* or telepatholog* or teleradiol* or tele radiol*).mp.

60 ((remote* or distance* or distant or online or virtual or telephone* or phone* or video* or internet* or computer* or sensor* or modem or webcam or website* or email or virtual or mobile or smartphone* or Application* or apps or technology or technologies or digital) adj7 (rehabilitation or therap* or treatment* or communication* or consult* or healthcare or health care or program or programs or programmes or programme or specialist* or counsel* or supervis* or care or support* or monitor* or virtual reality or virtual environment* or intervention*)).mp.

61 ((remote* or distance* or distant or online or virtual or telephone* or phone* or video* or internet* or computer* or sensor* or modem or webcam or website* or email or virtual or mobile or smartphone* or Application* or apps or digital or technology or technologies) adj7 (cognitive behavio?r or cbt or psychother*)).mp.

62 (mhealth or m health or mobile health).mp.

63 ((iphone? or i phone? or smartphone? or PDA or Personal Digital Assistant? or telephone or telephones or TRANSTELEPHON* or phone or phones) and (counselling or diagnose? or diagnosing or ((drug? or prescription? or disease? or outpatient? or inpatient? or patient?) adj3 (care or management or therap* or treat or treating or treatment?)) or (patient? adj2 education) or ((preventive or preventative) adj2 care) or public health or self care)).mp.

64 (e care or ecare or e consult* or econsult* or e diagnos* or ediagnosis* or e health* or ehealth* or e medicine or emedicine or e nurs* or enurs* or e physician? or ephysician? or e psych* or epsych* or e therap* or etherap*).mp.

65 (Mobile healthcare* or mobile health care* or computer mediated therap*).mp. (342)

66 ((health* or treat* or therap* or intervention* or assist* or selfmanag* or self manag*) adj6 (computer* or technolog* or software or digital)).mp.

67 (icbt or digital intervent* or digital care or online treatment* or online therap* or virtual care* or (digital adj2 (care? or tool?)) or self help* or selfhelp* or videoconferen* or video conference* or helpline* or help line* or self care or selfcare or self manage*).mp.

68 (virtual and day hospital*).mp.

69 (home* adj2 monitor*).mp.

70 ((online or virtual or digital) adj3 (communit* or forum* or discussion* or tool?)).mp.

71 (Technolog* adj3 tool?).mp.

72 exp Self Care/ or Self-Management/

73 (internet or virtual* or online or digital*).ti,kf.

74 (web adj7 (rehabilitation or therap* or treatment* or communication* or consult* or healthcare or health care or program or programs or programmes or programme or specialist* or counsel* or supervis* or care or support* or monitor* or virtual reality or virtual environment* or intervention*)).mp.

75 (App or apps or facetime* or helpline* or store-and-forward* or store-forward* or skype* or video* or zoom or webbased tool* or web-based tool* or voice-over or voiceover or VoIP).ti,kf.

76 (internet or virtual* or online or digital*).ti,ab,kf.

77 user-centered design/ or exp user-computer interface/ or video games/ or web browser/ or exp social media/

78 (cdrom or cd-rom or interactive computer or computer based or chatroom* or chat room*).mp.

79 smart.mp.

80 (web based or webbased).mp.

81 or/35-80

82 pc.fs. or prevent*.mp.

83 Primary Prevention/

84 exp Help-Seeking Behavior/

85 risk reduction.mp. or exp Risk Reduction Behavior/ or onset.mp.

86 exp Health Promotion/

87 exp Health Behavior/

88 exp Health Education/

89 (risk adj2 reduc*).mp.

90 intervention*.mp.

91 health promot*.mp.

92 healthy weight.mp.

93 body project.mp.

94 "image and mood".mp.

95 ((health or patient) adj2 educat*).mp.

96 (risk reduction or onset).mp.

97 or/82-96

98 27 and 81 and 97

99 34 or 98

100 adolescent/ or exp child/ or exp infant/

101 (child$ or adolescen$ or boy$ or girl$ or teen$ or schoolchild$ or preschool$ or pre-school$ or infant$ or baby or babies).mp.

102 (young person or young people or young adult*).mp.

103 (youth$ or juvenile$ or adolesc*).mp.

104 (infancy or newborn* or kid or kids or toddler*).mp.

105 (minor$ or pubert$ or pubescen$ or prepubescen$ or pediatric$ or paediatric$ or peadiatric$).mp.

106 or/100-105

107 (emerging adult* or college or colleges or university or universities or student or students).mp.

108 106 or 107

109 99 and 108

110 limit 99 to ("all child (0 to 18 years)" or "young adult (19 to 24 years)"

111 109 or 110
